# Incentive conflict and supply contracts under carbon cap policy

**DOI:** 10.1371/journal.pone.0277777

**Published:** 2022-11-17

**Authors:** Mithu Rani Kuiti, Preetam Basu, Debabrata Ghosh

**Affiliations:** 1 School of Management & Entrepreneurship, Indian Institute of Jodhpur, Karwar, Rajasthan, India; 2 Department of Analytics, Operations and Systems, Kent Business School, University of Kent, Canterbury, United Kingdom; 3 Strategy, Operations, and Entrepreneurship Group, Essex Business School, University of Essex, Colcheste, United Kingdom; Tohoku University, JAPAN

## Abstract

Environmental regulations, once promulgated, can cause incentive conflict between manufacturers and suppliers. A manufacturer facing the regulation may undertake choices that can affect his sourcing decisions with the supplier. To analyze this, we develop a game-theoretic model considering a manufacturer who faces a per-unit carbon emissions cap and sources from a supplier. The manufacturer operates in a carbon sensitive market. We analyze the responses of the manufacturer and supplier and show that since the burden of carbon emissions cap is borne by the manufacturer, the first-best outcomes are not reached. Therefore, the supplier may offer different contracts to incentivize the manufacturer. We study two mechanisms: the two-part tariff and the revenue-and-investment sharing contracts. We show how such contracts achieve coordination and deliver efficient supply chain outcomes. Interestingly, we find that the contract preferences of the manufacturer and the supplier may not be the same and vary under different market conditions. Summarily, we highlight important considerations for the supply chain players in designing suitable incentives.

## 1. Introduction

Environmental pollution and global warming have been key concerns for nations over the last few decades. The primary focus within this has been the reduction of greenhouse gases (GHGs). Different countries have promulgated policies to reduce GHG emissions, which have had a significant impact on firms’ products and supply chain processes. Consequently, environmental improvements in supply chains have received attention from firms in addition to the design and development of green products [1]. Scholars as well, have explored problems in the areas of product life cycle management, carbon footprint measurement, reverse logistics, remanufacturing etc. ([2–5] and the references therein). Our paper considers an incentive-conflict problem arising from the carbon cap regulation and undertakes a multi-stakeholder perspective.

While studies have analyzed the choice of optimal environmental regulations and examined the decisions of the government as a social planner [6, 7], few have examined how such environmental regulations, once enacted, can create incentive conflict between supply chain entities. Consider, for example, the Environmental Protection Agency’s (EPA’s) regulations in the U.S. for lowering nitrogen-oxide emissions from commercial trucks, cement mixers and trash trucks that propose tighter emission norms from the model year 2027. The regulations require manufacturers to design better exhaust systems for gasoline and diesel engine models. This entails new technology investments and supplier relationships. In response to the proposed changes to the emission norms, the Owner-Operator Independent Drivers Association in the U.S. said that the costs of compliance with such norms would be high and much of these costs would be passed on to the customers. The Association also mentioned that the requisite technology is not available at lower costs as ideally expected and manufacturers may struggle to develop a complying product (https://www.wsj.com/articles/epa-aims-to-cut-toxic-emissions-from-commercial-trucks-11646670626?page=1).

For several countries such as France and Germany, the Covid-19 bailout packages were closely tied up with the pressure on automakers to adopt sustainability measures in their supply chains. This meant an opportunity to collaborate with domestic or nearshore suppliers or select suppliers with the best records on emissions (https://www.forbes.com/sites/oliverwyman/2020/05/27/mid-covid-auto-must-face-its-next-challenge-carbon-neutrality/?sh=3f6b681656ee). Regulatory changes, therefore, create substantial pressure on manufacturers who may undertake decisions that can affect their relationship with their suppliers. This necessitates the investigation of a manufacturer’s and supplier’s decisions in a supply chain under environmental regulations. Furthermore, considering the additional costs or penalties that the manufacturer has to bear, opportunities for cost-sharing and collaboration between the manufacturer and supplier arise to help comply with the environmental norms. This necessitates the analysis of contracts that can benefit the players and the supply chain.

We, therefore, raise the following research questions—*1) How does an environmental regulation such as the carbon cap impact a manufacturer-supplier dyad*? *2) Are there supply contracts that enable higher supply chain performance under the carbon cap regulation*? To answer these questions, we analyze a manufacturer-supplier dyad where the manufacturer faces carbon cap regulation and undertakes greening as a response. The manufacturer sources raw material from the supplier and operates in a green-sensitive market. In a game-theoretic setting, we analyze the decisions of each player. We explore mechanisms to align the incentives of the players in the supply chain and study two mechanisms which implement the first-best outcomes. We conduct a comparative analysis to identify various conditions under which each player would prefer a specific contract. We derive several policy insights and lessons for supply chain managers.

### 1.1 Summary of findings

Our results show that the manufacturer strategically balances the pricing and the carbon emission decisions under different scenarios based on varying levels of consumer green sensitivity, price elasticity and green investment expenditures. As the carbon emission standards are tightened, the manufacturer is forced to increase the amount of carbon reduction. This also results in an increase in prices by the supplier and manufacturer respectively. Therefore, stricter carbon emission standards force the manufacturer to increase the amount of carbon reduction and selling price. However, with an increase in price, consumer welfare is affected. These findings have important ramifications for policymakers who may subsidize carbon reduction initiatives of manufacturers leading to improved consumer welfare.

We explore contractual agreements through which the supplier may share some of the manufacturer’s cost of greening. We find that based on the market characteristics and greening costs, the contract preference of the supplier and the manufacturer may differ. Under higher greening costs or high price sensitivity, the manufacturer prefers a revenue-and-investment sharing contract whereas, if the greening cost is low or price sensitivity is low, the manufacturer prefers a two-part tariff contract. Additionally, the manufacturer prefers the two-part tariff contract when consumers have a high carbon reduction sensitivity, while a revenue-and-investment sharing contract as consumers’ carbon reduction sensitivity decreases. The above results provide important directions for the supply chain players to design suitable incentives that can drive effective win-win outcomes.

The rest of the paper is organized as follows. In the following section, we discuss the background motivation of our study. Section 3 discusses the relevant literature. Section 4 discusses the model conceptualization and formulation. The analytical results are presented in Sections 4 and 5. The supply contracts and numerical results are discussed in Section 6. Section 7 concludes the paper and presents future research ideas.

## 2. Background

Our study is motivated by the environmental regulations enacted in different geographies and noticeably how they impact manufacturers and their suppliers. We discuss two such regulations. The Corporate Average Fuel Economy (CAFE) and Greenhouse Gas emission (GHG) regulations that were jointly issued by the National Highway Traffic Safety Administration (NHTSA) and Environmental Protection Agency (EPA) in the U.S., underwent a significant change to improve fuel efficiency and reduce carbon emissions for passenger cars and light trucks(https://www.wsj.com/articles/new-fuel-efficiency-standards-risk-splitting-groups-11627909200). The new norms have provisions that force vehicle manufacturers to review their existing product designs and consider changes to adhere to the government-mandated carbon emission standards. By extension, this has implications for their suppliers since product design changes require suppliers to work towards new component manufacturing.

Similarly, in the EU, in the road transport sector, the EU legislators fixed the emission targets for light-duty (cars and vans) and heavy-duty vehicles (HDV) (buses and trucks) to reduce GHG emissions in the transport sector. The legislation mandates that the manufacturer pay the excess emissions premium for each registered light-duty vehicle if the manufacturer’s fleet does not meet the set targets. Such regulatory changes combined with the Covid-19 impact on the automakers due to semiconductor shortages mean that suppliers work with extremely thin margins, manufacturing disruptions, and higher costs of components (https://www.wsj.com/articles/it-was-a-pretty-good-year-in-the-car-businessexcept-for-suppliers-11638700201). There arises a need to assess the supply chain decisions under environmental norms and examine contracts that can drive a win-win outcome for the supply chain entities while complying with the environmental norms. This motivates our study.

Lastly, our study also considers the demand-side effect of environmental initiatives. We consider that firms (namely, the manufacturer and supplier) operate in a market where consumers are environmentally friendly. In the last few decades, consumers’ preferences for green products have led to additional implications for firms. Studies have shown that there are consumer segments that are environmentally conscious and prefer green products. For many firms, this signals an opportunity to drive product changes and target such consumer markets [8–10]. We, therefore, consider consumer demand to be positively affected by the level of the greenness of a product. An eco-friendly or green product by definition is one, which consumes fewer resources during production and emits less carbon [2, 11, 12]. Therefore, we consider a model of consumer demand, which depends on the product’s green level, which is measured by reduced carbon emissions. The higher the reduction in carbon emissions, the greener the product.

Our study, therefore, aims to develop an integrated model of decision-making considering consumer preferences, and firms’ decisions in a supply chain. We next discuss the background literature and position our work in the extant literature.

## 3. Literature review

Our work spans two streams of literature: a) studies based on carbon emission policies and b) supply chain coordination in presence of product’s greenness. In what follows, we discuss some of the relevant literature in the context of our current work.

### 3.1 Studies based on carbon emission policies

Extant studies have examined important issues related to carbon emissions in supply chains [13–16]. A notable number of studies [17–20] have analyzed firm decisions in the presence of various kinds of government-mandated carbon emission policies. For example, Chen et al. [21] derived the optimal tax in a supply chain that not only meets the carbon emission reduction target but also achieves sustainable economic development goals. It has been observed that the implementation of a carbon tax in the supply chain motivates manufacturers to invest in carbon reduction technology [22, 23]. Another popular carbon emission policy—cap-and-trade has also been studied in the supply chain context [24–26]. Tong et al. [27] showed that the cap-and-trade policy and consumers’ preference for low-carbon product jointly stimulates the manufacturer’s and retailer’s behavior toward sustainable practices. Later, Wang and Wu [28] showed how cap-and-trade policy plays a role in strategic decisions of carbon emission reduction and collection of used products in a closed-loop supply chain. The authors found that the carbon emission abatement level increases with the carbon trading price, whereas the used product return rate decreases with the carbon trading price. In addition, literature has also examined competition in supply chains under carbon emissions [29–31]. Zhou et al. [32] showed that implementation of carbon tax policy in a supply chain not only enhances the sales for the retailer of environment-friendly products relative to other competing retailers but also improves social welfare.

Other areas such as technology investments in the presence of carbon tax [22], third-party logistics as a service [33], and interconnectedness of delivery time and carbon emission in presence of carbon tax [34] etc. have been discussed in the literature. However, carbon-cap as a regulation and supply chain decisions have had limited focus. Secondly, the incentive conflict that arises from carbon-cap regulation in supply chains has not been studied. For instance, the manufacturer has to mandatorily meet the carbon-cap regulation which leads to a higher cost burden for the manufacturer and can lead to an incentive conflict in the supply chain. Extant literature does not, however, highlight incentive conflicts that may arise from carbon emission regulations. We study the problem considering the manufacturer-supplier relationship where the manufacturer must bear the unit carbon emission cap mandated by the government. We examine how the amount of reduced carbon emission, prices, and profits are influenced by the regulation. Incentive conflict problems under carbon cap policies have received limited attention in the past and this is the gap we seek to address.

### 3.2 Supply chain coordination in presence of product’s greenness

In this section, we review literature that considers supply chain coordination in sustainability settings. Swami and Shah [35] and Bai et al., [36] explore game theoretic models where demand depends on the level of green innovation and propose various coordination models. The studies however, analyze manufacturer-retailer dyads and not manufacturer-supplier dyads as we study in this paper. Different sets of issues underlie manufacturer-supplier relationships as the manufacturer incurs manufacturing costs and responds to the carbon-cap regulation whereas, the supplier undertakes decisions on raw material components that may influence the manufacturer’s pricing.

Supply chain coordination literature has studied various contracts such as revenue sharing [37], cost-sharing [38], two-part tariff [12, 16], revenue and investment sharing [35]. As regulations evolved, several of these contracts were examined in the sustainable supply chain context as well. For example, Yi and Li [39] designed the cost-sharing contract for energy saving and emissions reduction in the supply chain in presence of Government subsidy and carbon tax. Xu et al. [25] analyzed the revenue sharing and two-part tariff contracts for a two-echelon supply chain consisting of manufacturer-retailer in the presence of a cap-and-trade policy. Further, Bai et al. [40] showed that the economic and environmental sustainability of the decentralized system can be improved under revenue and cost-sharing contract in the presence of a cap-and-trade policy.

Several coordination mechanisms have been studied in extant OM literature such as the revenue-sharing, wholesale price, quantity flexibility, price-discount, and sales-rebate contracts. However, models considering carbon regulations and conflict arising from green initiatives are still nascent. At the time of writing, issues related to impact of carbon taxes and carbon pricing were discussed at the Conference of Parties (COP26) Summit, under the aegis of United Nations Framework Convention on Climate Change (UNFCCC) at Glasgow (https://iccwbo.org/media-wall/news-speeches/global-business-tells-governments-at-cop26-put-a-price-on-carbon-but-do-it-the-right-way/), demonstrating the need for improved understanding on how regulations affect supply chain entities. Limited studies have analyzed such incentive conflict and coordination problems in manufacturer-supplier dyads. Our paper seeks to address this gap.

Below we provide a summary of relevant literature and position our current work in the extant literature (refer to Table 1).

**Table 1:** Summary of literature review.

Papers	Supply Chain Structure	Players Effort in	Demand is a function of	Carbon emission policy	Contracts
Swami and Shah [15]	Manufacturer and Retailer	Greening	Price, Manufacturer’s and Retailer’s Greening Effort (Single Product)	No	TT
Ghosh and Shah [11, 41]	Manufacturer and Retailer	Greening	Price and Greening Effort (Single Product)	No	TT, CS
Xu et al. [25]	Supplier and manufacturer	Greening	price and Greening	Cap-and -trade	TT and RS
Ma et al. [38]	Two Manufacturers and Retailer	Greening	Price, Substitute Product’s Price and Greening Effort	No	CS
Raj et al. [37]	Manufacturer and Retailer	Greening, CSR	Price, Greening Effort and CSR	No	TT, RS, CS, RGCS
Yi and Li [39]	Manufacturer and Retailer	Greening & energy saving	Price, energy saving and Carbon emission level	Carbon Tax	CS
Bai et al. [36]	Supplier and Manufacturer	Greening	Price, Substitute Product’s Price and Greening Effort	Cap-and-trade	RIS
Chen et al. [21]	Manufacturer and Retailer	Greening	price	Carbon Tax	—
Ghosh et al. [42]	Manufacturer and Retailer	Greening	Price	No	CS
Bai et al. [22]	Manufacturer and Retailer	Greening and promotion	Price, greening and promotion effort	Carbon tax	TT, RS
Kuiti et al. [24]	Manufacturer and Retailer	Greening, CSR	Price, Complementary Product’s Price, Greening Effort and CSR	Cap-and -trade	TT, CS, ES
He et al. [34]	Manufacturer and Retailer	Greening and Delivery	Price, Greening and Delivery time	Carbon Tax	TWCS
Bai et al. [40]	Manufacturer and Retailer	CSR and Greening Effort	Price, Cross Price, Greening and CSR	Cap-and-Trade	RS, CS
Our Paper	Supplier and Manufacturer	Greening	Price, amount of Carbon reduction	Carbon Cap	TT, RIS

**Note:** CSR: Corporate Social Responsibility; TT: Two-part Tariff; RS: Revenue Sharing; CS: Cost Sharing; RIS: Revenue Investment Sharing; RGCS: Revenue Greening Cost Sharing; ES: Effort Sharing, TWCS: Two Way Cost Sharing

## 4. Problem description

We begin with the model preliminaries. We consider a supply chain consisting of a supplier and a manufacturer. The manufacturer produces the product and directly sells it to end customers after procuring raw material from the supplier. Since the manufacturer is primarily responsible for the product and the associated carbon emissions with it, it is assumed that the government-mandated carbon emissions cap applies to the manufacturer. The manufacturer incurs the cost of greening even though both supply chain players benefit from the green sensitive consumer demand leading to an incentive conflict. Further, the manufacturer undertakes greening investments to redesign the product to meet government-mandated emission norms [26, 41].

### 4.1 Demand model

The product’s demand is assumed to be a linear function of the carbon emission reduction amount (Δ*k*) and selling price of the product (*p*). Linearity assumptions with respect to price and non-price variables in supply chain settings involving inter-firm interactions have been made in several operations management and marketing studies [24, 40, 43]. In our model, we assume that the initiative for the reduction of a product’s carbon emission is considered a green product innovation. The demand function therefore, reflects a ‘green’ conscious consumer.

In keeping with the current trend in sustainable consumption, studies have indicated that the environmental awareness of consumers has a positive impact on their buying decisions [44]. Empirical studies have shown that consumers prefer to buy products from companies behaving in environmentally responsible manner [45] and they refuse to buy products from those who are found accused of being polluters [46]. From these findings, we assume that product greening level has a positive impact on demand and this effectiveness is measured by (*γ*) in our model. Since, our model is motivated by the fuel efficiency changes in vehicles under various carbon cap regulations, fuel efficiency has a direct implication on consumer demand [47]. Therefore, the product’s demand is considered to be a function of price and carbon emission reduction amount [35, 48, 49] given as,

$$D\left(p,\Delta k\right)=\alpha -\beta p+\gamma \Delta k$$ (1)

The demand function (1) is linearly increasing in Δ*k*, the amount of reduction of carbon emission per unit and decreasing in *p*, the selling price of the product. All model parameters and decision variables are provided in Table 2 for reference.

**Table 2:** Parameters and notations.

Notation	Description
For the manufacturer:	
*p*	Manufacturer’s per unit selling price of a product **(decision variable).**
Δ*k*	Per unit reduction of carbon emission **(decision variable).**
*k*_0_	Initial per unit carbon emission.
*k*_1_	Carbon emission after reduction.
*N*_1_	Carbon cap on per unit product mandated by the government.
*α*	Base market demand of a product.
*β*	Price effect on market demand.
*γ*	Carbon emission reduction effect on market demand.
*c*_*m*_	Manufacturer’s cost of production.
*τ*	The marginal cost of carbon emission.
*I*	The coefficient of investment in a new technology to reduce the carbon emission.
$\bar{I}$	The upper limit of the coefficient of fixed investment.
$\hat{I}$	The lower bound on the coefficient of fixed investment.
*D*(*p*,Δ*k*)	The demand function.
Π_*M*_ (*p*, Δ*k*)	Manufacturer’s profit in the decentralized channel setting
∏Mttp,Δk	Manufacturer’s profit in the two-part tariff contract setting.
∏SRISw	Manufacturer’s profit under the revenue and investment-sharing contract
For the supplier:	
*w*	Supplier’s wholesale price of the raw material **(decision variable).**
*c*_*s*_	Supplier’s unit cost of raw material.
*F*	Fixed cost charged by the supplier in the two-part tariff contract.
Π_*S*_ (*w*)	Supplier’s profit in the decentralized channel setting
∏Sttw	Supplier’s profit in the two-part tariff contract setting.
∏MRISp,Δk	Supplier’s profit under the revenue and investment-sharing contract
For the supply chain	
Π_*C*_ (*p*, Δ*k*)	Total supply chain’s profit in the centralized system.
Π_*DC*_ (*p*, Δ*k*)	Total supply chain’s profit in the decentralized system.

### 4.2 Cost functions

Let *k*_0_ be the initial per unit carbon emission and Δ*k* be the amount of reduced carbon emission per unit achieved by the manufacturer. Thus, the final per unit carbon emission level reached is given by *k*_1_ = *k*_0_ - Δ*k*. For non-trivial cases, we assume that the amount of carbon emission reduction of a product (Δ*k*) should satisfy 0 ≤ Δ*k* ≤ *k*_0_. We further assume that the manufacturer incurs a higher fixed and marginal cost for improving the product’s greenness. The improvement of product’s greenness indicates the reformation of product attributes when incorporated, thus, making the older product obsolete [50]. Therefore, in our model, we consider an increasing and convex fixed cost structure for improvement in product’s greenness. The manufacturer’s fixed investment for greening of a product is given by *I*(Δ*k*)^2^ where *I* is the investment coefficient [11, 41]. Such investments are large and therefore, we assume that the value of the investment coefficient should be greater than $\frac{B_{1}^{2}}{4\beta }$ where *B*_1_ = *γ* – *βτ* and *τ* is the manufacturer’s marginal cost of carbon reduction. Furthermore, the parameters should satisfy $\tau \leq \frac{\gamma }{\beta }$ and *α* ≥ *β*(*c*_*m*_ + *c*_*s*_) for non-negative values of selling price and the amount of reduction of carbon emission per unit, respectively.

### 4.3 Carbon cap

To model government-mandated carbon cap, it is assumed that the manufacturer has a carbon emission cap *N*_1_ on per unit production quantity. Thus, the manufacturer’s final carbon emission level is assumed to satisfy *k*_1_ ≤ *N*_1_. Since *k*_1_ = *k*_0_ - Δ*k*, we have Δ*k* ≥ *k*_0_—*N*_1_.

#### 4.3.1 Profit functions

Based on the above description, the profit expressions of the manufacturer and the supplier in the decentralized channel are formulated below. Since, the manufacturer produces the product by procuring raw material from the supplier, we consider a Stackelberg model where the supplier determines the wholesale price of the raw material first, followed by the manufacturer’s decisions [17]. In our model, under government-mandated carbon emission cap, the manufacturer determines the selling price and the amount of carbon reduction per unit that needs to be achieved. The manufacturer’s profit function under government-mandated carbon emission cap is given by,

$$\begin{array}{l} \prod_{M}\left(p,\Delta k\right)=\left(p-c_{m}-w-\tau \left(\Delta k\right)\right)D\left(p,\Delta k\right)-I{\left(\Delta k\right)}^{2}\\ \;s.t.,\Delta k\geq k_{0}-N_{1} \end{array}$$ (2)

The supplier’s profit function is given by,

$$\prod_{S}\left(w\right)=\left(w-c_{s}\right)D\left(p,\Delta k\right)$$ (3)

As a benchmark case, we evaluate the first-best or the centralized supply chain decisions. The profit of the centralized chain is given by,

$$\begin{array}{l} \prod_{C}\left(p,\Delta k\right)=\left(p-c_{m}-c_{s}-\tau \left(\Delta k\right)\right)D\left(p,\Delta k\right)-I{\left(\Delta k\right)}^{2}\\ \;s.t.,\Delta k\geq k_{0}-N_{1} \end{array}$$ (4)

Below we present the equilibrium solutions for the decentralized and centralized cases (Lemmas 1 and 2 respectively). All proofs are provided in the S1 Appendix.

**Lemma 1:** *The carbon emission level for the decentralized case*, *at equilibrium*, *achieved by the manufacturer after carbon emission reduction*, *under government-mandated per unit emission*, *is given by*,

$$k_{1}^{*}=\left\{\begin{array}{ccc} 0 & \text{when} & I\leq \hat{I}\\ k_{0}-\Delta k^{*} & \text{when} & \hat{I}<I<\bar{I}\\ k_{0}-N_{1} & \text{when} & I=\bar{I} \end{array}\right.$$

*The equilibrium wholesale price set by the supplier is given by*,

$$w_{1}^{*}=\frac{1}{2\beta }\left\{\begin{array}{ccc} \left[\alpha -\beta \left(c_{m}-c_{s}\right)+B_{1}k_{0}\right] & \text{when} & I\leq \hat{I}\\ \left[\alpha -\beta \left(c_{m}-c_{s}\right)\right] & \text{when} & \hat{I}<I<\bar{I}\\ \left[\alpha -\beta \left(c_{m}-c_{s}\right)+B_{1}\left(k_{0}-N_{1}\right)\right] & \text{when} & I=\bar{I} \end{array}\right.$$

*The equilibrium selling price of the product set by the manufacturer is given by*,

$$p^{•}=\frac{1}{4\beta }\left\{\begin{array}{ccc} 3\alpha +\beta \left(c_{m}+c_{s}\right)+\left(3\gamma +\beta \tau \right)k_{0} & \text{when} & I\leq \hat{I}\\ \frac{1}{4\beta I-B_{1}^{2}}\left[4\beta I\left(3\alpha +\beta \left(c_{m}+c_{s}\right)\right)+2B_{1}\gamma \left(\alpha -\beta \left(c_{m}-c_{s}\right)\right)-4B_{1}^{2}\alpha \right] & \text{when} & \hat{I}<I<\bar{I}\\ 3\alpha +\beta \left(c_{m}+c_{s}\right)+\left(3\gamma +\beta \tau \right)\left(k_{0}-N_{1}\right) & \text{when} & I=\bar{I} \end{array}\right.$$

Where $\Delta k^{*}=\frac{B_{1}}{2\left(4\beta I-B_{1}^{2}\right)}\left[\alpha -\beta \left(c_{m}+c_{s}\right)\right]$, $\bar{I}=\frac{B_{1}}{8\beta \left(k_{0}-N_{1}\right)}\left[\alpha -\beta \left(c_{m}+c_{s}\right)+2B_{1}\left(k_{0}-N_{1}\right)\right]$ and $\hat{I}=\frac{B_{1}}{8\beta k_{0}}\left[\alpha -\beta \left(c_{m}+c_{s}\right)+2B_{1}k_{0}\right]$. All the solutions and corresponding profit function in different cases are given in S1 Table in S1 Appendix.

In the above lemma, the first and second conditions (i.e., $I\leq \hat{I}$ and $\hat{I}<I<\bar{I}$) are termed as Case 1 and the condition (i.e., $I=\bar{I}$) is termed as Case 2. Under Case 1, the manufacturer’s per unit reduction in carbon emission is given by $Min\left\{k_{0},\Delta k^{*}\right\}$. We observe that when the investment coefficient is less than or equal to the threshold investment coefficient for zero emission, i.e., $I\leq \hat{I}$, the per unit reduction in carbon emission is Δk* = *k*_0_ which means that the amount of carbon reduction achieved by the manufacturer is equal to the total current per unit carbon emission. We call this region a zero-emission region and denote $\hat{I}$ as the *threshold investment coefficient for zero-emission*. This is feasible because the greening investment coefficient is lower than the bound given by $\hat{I}$.

When $\hat{I}<I<\bar{I}$under Case 1, the per unit reduction in carbon emission is Δk*, *i*.*e*., the reduced per unit emission achieved is, $k_{1}^{*}=k_{0}-\Delta k^{*}$. We call this the *optimal emission case*. Lastly, when $I=\bar{I},$ the required fixed investment in green technology of the manufacturer is highest and the reduced carbon emission is Δk* = *k*_0_ –*N*_1_ which implies that the per unit carbon emission is equal to *N*_1_, the government-mandated cap. We call $\bar{I}$as the *benchmark investment coefficient for government-mandated emissions*. The equilibrium wholesale and selling prices vary with greening investment under different cases as derived above.

In the centralized case,

**Lemma 2:** *The optimal carbon emission level achieved by the manufacturer after carbon emission reduction*, *under government-mandated per unit emission*, *is given by*,

$$k_{1}^{C}=\left\{\begin{array}{ccc} 0 & \text{when} & I\leq \hat{I}\\ k_{0}-\Delta k_{C} & \text{when} & \hat{I}<I<\bar{I}\\ k_{0}-N_{1} & \text{when} & I=\bar{I} \end{array}\right.$$

*The optimal selling price of the product set by the centralized decision maker is given by*,

$$p_{C}=\frac{1}{2\beta }\left\{\begin{array}{ccc} \alpha +\beta \left(c_{m}+c_{s}\right)+\left(\gamma +\beta \tau \right)k_{0} & \text{when} & I\leq \hat{I}\\ \frac{1}{4\beta I-B_{1}^{2}}\left[4\beta I\left(\alpha +\beta \left(c_{m}+c_{s}\right)\right)+2B_{1}\gamma \left(\alpha -\beta \left(c_{m}-c_{s}\right)\right)-4B_{1}^{2}\alpha \right] & \text{when} & \hat{I}<I<\bar{I}\\ \alpha +\beta \left(c_{m}+c_{s}\right)+\left(\gamma +\beta \tau \right)\left(k_{0}-N_{1}\right) & \text{when} & I=\bar{I} \end{array}\right.$$

Where $\Delta k_{C}=\frac{B_{1}}{4\beta I-B_{1}^{2}}\left[\alpha -\beta \left(c_{m}+w\right)\right]$

$$\bar{I}=\frac{B_{1}}{4\beta \left(k_{0}-N_{1}\right)}\left[\alpha -\beta \left(c_{m}+c_{s}\right)+B_{1}\left(k_{0}-N_{1}\right)\right]\;\text{and}\;\hat{I}=\frac{B_{1}}{4\beta k_{0}}\left[\alpha -\beta \left(c_{m}+c_{s}\right)+B_{1}k_{0}\right].$$

We analyze the above results in the following section. The optimal emission case and zero emission case in Case 1 are represented by the subscript "11" and "12" respectively (e.g., *P*_11_,*P*_12_ etc.), and the subscript "2" denotes Case 2. All the solutions and the corresponding profit functions in different cases are given in the S2 Table in S1 Appendix.

## 5. Results and analysis

In this section, we discuss several implications of the results derived above. The equilibrium values of the selling price, reduced carbon emission and the wholesale price are compared across both the cases (Case 1 and Case 2). We further analyze the impact of various parameters on the decision variables. Different mechanisms to coordinate the dyadic supply chain are also presented.

### 5.1 Comparative statics

**Proposition 1**:

- *The per unit selling price of the product increases with the increase in carbon reduction elasticity i*.*e*., (i) $\frac{\partial }{\partial \gamma }\left(p_{11}\right)\geq 0,\frac{\partial }{\partial \gamma }\left(p_{12}\right)\geq 0$, (ii) $\frac{\partial }{\partial \gamma }\left(p_{2}\right)\geq 0.$
- *The per unit reduction in carbon emission of the product decreases with the increase in price sensitivity*, *i*.*e*.,
  - $\frac{\partial }{\partial \beta }\left(\Delta k_{11}\right)\leq 0.$

Proposition 1(a) illustrates the cross effect of carbon reduction elasticity on the optimal pricing decision of the manufacturer. It is observed that the manufacturer strategically increases the price of the product in markets where consumers have a higher preference for greenness. The result suggests that manufacturers can extract a price premium from green sensitive consumers. This is supported by our observations earlier, where to support manufacturers in designing and developing environmentally friendly products, the EU follows a strategy of *CO*_2_ labeling of cars, which provides key information about fuel efficiency and *CO*_2_ emissions to new customers. With the aim to drive carbon reduction initiatives, while policy makers formulate emission norms, the above results also demonstrate that policy support is required to create green sensitive consumer markets. Under the twin effects of carbon emission norms and green markets, higher reduction in carbon emissions of products can be achieved.

Interestingly, proposition 1(b) reveals that for a price sensitive consumer segment, the manufacturer can strategically reduce the amount of carbon reduction of the product. Eq (2) highlights that the manufacturer maintains his profits by reducing associated cost related to greenness of the product (both variable and fixed cost of greening). Thus, in price sensitive consumer markets, manufacturers are unable to provide higher level of carbon reduction. We next study the effect of carbon reduction cost on the equilibrium decisions.

**Proposition 2:** *The per unit reduction in carbon emission and the manufacturer’s selling price decrease with the increase of fixed investment cost under Case1*, *i*.*e*.,

- *(i)* $\frac{\partial \Delta k_{11}}{\partial I}\leq 0$ *and (ii)* $\frac{\partial p_{11}}{\partial I}\leq 0.$

The result shows that with increase in fixed investment cost, the manufacturer reduces the amount of carbon emission reduction. Interestingly though, the price of the product is also reduced with increase in investment cost. Eq (2) highlights that the manufacturer influences the market demand through two decisions under increasing investment cost. While he strategically reduces the amount of carbon reduction, he also reduces the price to increase the market demand, thus, maintaining his profits.

Next, we analyze the impact of government-mandated carbon cap on the strategic decisions of the players.

**Proposition 3:** *The impact of per unit carbon cap on the strategic decisions of players under case 2 is as follows: (i) $\frac{\partial \Delta k_{2}}{\partial N_{1}}\leq 0$, (ii) $\frac{\partial w_{2}}{\partial N_{1}}\leq 0$, and (ii) $\frac{\partial p_{2}}{\partial N_{1}}\leq 0$.*

The above result demonstrates the critical role that government-mandated standards play in forcing firms to undertake carbon reduction initiatives. We observe that as carbon emission standards are tightened (i.e., the value of carbon cap *N*_1_ decreases), the manufacturer is forced to increase the amount of carbon reduction. This also results in increase in prices by the supplier and manufacturer respectively. This is an outcome of the strategic decision of the players to maintain their respective margins. The result shows why policy makers have an important role to play in forcing manufacturers to undertake carbon reduction initiatives, particularly, when voluntary initiatives by firms are not sufficient to achieve higher greening standards. However, consumer welfare is affected since the selling price increases with tighter cap. Policy makers may therefore consider subsidizing carbon emission initiatives of manufacturers, or supply chain entities may consider sharing the cost of greening under government norms.

**Proposition 4:** *The optimal total profits of the centralized and decentralized channels satisfy the following relations*:

$$\prod_{C}\geq \frac{4}{3}\prod {}_{DC}\text{when}\;I\leq \hat{I}$$

$$\prod_{C}=\frac{4}{3}\prod {}_{DC}\;\text{when}\;\hat{I}\leq I\leq \bar{I},$$

$$\prod_{C}\geq \frac{4}{3}\prod {}_{DC}\;\text{when}\;I\geq \bar{I},$$

The above result suggests that the centralized channel profit is always greater than the decentralized channel. Clearly, coordination between the supplier and manufacturer in the decentralized channel can reduce this profit differential. Since the burden of the carbon emission cap as mandated by the Government falls on the manufacturer, the supplier could offer a different contract to increase the profitability of both channel members. In what follows, we propose a two-part tariff and a revenue and investment-sharing contract to study channel coordination (Proof is given in Appendix G in S1 Appendix).

## 6. Contract analysis

In this section, we analyze two different contracts in the context of government-mandated carbon cap. We describe a two-part tariff contract and a revenue sharing and investment-sharing contract to coordinate the channel.

### 6.1 Coordination with a two-part tariff contract

The two-part tariff contract is a widely useful mechanism to coordinate a supply chain [15, 26, 36]. However, few studies have focused on analyzing the two-part tariff contract in coordinating a carbon emission cap-based supply chain. In our problem, the supplier proposes a lower per unit raw material price *w*_*tt*_ and a fixed cost *F*_*tt*_ to the manufacturer. If the manufacturer accepts the contract, the profit functions of the manufacturer and supplier can be written as,

$$\begin{array}{l} \prod_{M/tt}\left(p,\Delta k\right)=\left(p-c_{m}-w_{tt}-\tau \Delta k\right)(\alpha -\;\beta p\text{+}\gamma \Delta \text{k})-I{\left(\Delta k\right)}^{2}-F_{tt}\\ \;s.t.,\;\Delta k\geq k_{0}-N_{1} \end{array}$$ (5)

$$\prod_{S/tt}\left(w\right)=\left(w_{tt}-c_{s}\right)\left(\alpha -\beta p+\gamma \Delta k\right)+F_{tt}$$ (6)

**Proposition 5:** *In the optimal emission case, the two-part tariff contract between the supplier and manufacturer coordinates the channel when $w_{tt}=c_{s}$ and satisfies the following*

$$\begin{array}{ccc} \frac{1}{8\beta }{\left[\alpha -\beta \left(c_{m}+c_{s}\right)+B_{1}k_{0}\right]}^{2}\leq F_{tt}\leq \frac{3}{16\beta }{\left[\alpha -\beta \left(c_{m}+c_{s}\right)+B_{1}k_{0}\right]}^{2} & \text{when} & I\leq \hat{I},\\ \frac{I}{2}\frac{{\left[\alpha -\beta \left(c_{m}+c_{s}\right)\right]}^{2}}{\left(4\beta I-B_{1}^{2}\right)}\leq F_{tt}\leq \frac{3I}{4}\frac{{\left[\alpha -\beta \left(c_{m}+c_{s}\right)\right]}^{2}}{\left(4\beta I-B_{1}^{2}\right)} & \text{when} & \hat{I}<I<\bar{I},\\ \frac{1}{8\beta }{\left[\alpha -\beta \left(c_{m}+c_{s}\right)+B_{1}\left(k_{0}-N_{1}\right)\right]}^{2}\leq F_{tt}\leq \frac{3}{16\beta }{\left[\alpha -\beta \left(c_{m}+c_{s}\right)+B_{1}\left(k_{0}-N_{1}\right)\right]}^{2} & \text{when} & I=\bar{I}. \end{array}$$

Furthermore, $\prod_{M/tt}\left(p_{tt},\Delta k_{tt}\right)+\prod_{S/tt}\left(w_{tt}\right)=\prod_{C}\left(p_{C},\Delta k_{C}\right)$

The above result indicates that the total supply chain profit under the two-part tariff contract is equal to the profit of the centralized channel. Thus, the two-part tariff contract perfectly coordinates the decentralized channel within a certain range of fixed cost paid by the supplier. Under this contract, the per unit charge of raw material by the supplier is sufficient to cover the per unit supplier cost *c*_*s*_. Thus, the contract incentivizes the manufacturer to lower his product price. Further, the supplier improves his profit from the fixed-fee component, which would be a result of negotiation between the supplier and manufacturer, within the limits provided by our result. Thus, both the supplier and manufacturer benefit under the two-part tariff contract.

In a two-part tariff contract, one of the parties commits to pay a fixed amount to the other along with the wholesale price. However, the applicability of the two-part tariff contract is a challenge because the supplier may not be willing to reduce the wholesale prices and make it close to per unit production cost. On the contrary, revenue and investment sharing contract would be applicable in scenarios where information sharing and financial reporting is transparent between the channel members. Thus we study the revenue and investment sharing in the next section.

### 6.2 Coordination with a revenue-and investment-sharing contract

In this contract, the supplier (leader of this strategic game) charges lower wholesale price (*w*_*ris*_) to the manufacturer (follower) and shares (1-*φ*) proportion of manufacturer’s fixed cost as well as per unit cost related to improvement of product’s greenness. In place of the above cost sharing, the manufacturer returns (1-*φ*) proportion of his revenue generated from selling the product to the supplier. The contract structure therefore involves both a revenue sharing and cost sharing mechanism involving the supply chain entities. The profit function of the manufacturer and supplier are therefore given by,

$$\begin{array}{l} \prod_{M/RIS}\left(p,\Delta k\right)=\left(\varphi p-c_{m}-w_{tt}-\varphi \tau \Delta k\right)(\alpha -\;\beta p\text{+}\gamma \Delta \text{k})-\varphi I{\left(\Delta k\right)}^{2}\\ s.t.,\;\Delta k\geq k_{0}-N_{1} \end{array}$$ (7)

$$\prod_{S/RIS}\left(w\right)=\left[w_{ris}-c_{s}+\left(1-\varphi \right)p-\left(1-\varphi \right)\tau \Delta k\right]D\left(p,\Delta k\right)-\left(1-\varphi \right)I{\left(\Delta k\right)}^{2}$$ (8)

Solving Eq (7) under contract coordination condition, we get the following result.

**Proposition 6:** *The revenue and investment-sharing contract between the manufacturer and supplier coordinates the channel in the optimal emission case when satisfying the following*

- $w_{rie}=\varphi \;c_{s}-\left(1-\varphi \right)c_{m}$.
- *φ satisfies* $\begin{array}{ccc} \frac{\frac{1}{16\beta }{\left[\alpha -\beta \left(c_{m}+c_{s}\right)+B_{1}k_{0}\right]}^{2}-Ik_{0}^{2}}{\frac{1}{4\beta }{\left[\alpha -\beta \left(c_{m}+c_{s}\right)+B_{1}k_{0}\right]}^{2}-Ik_{0}^{2}}\leq \varphi \leq \frac{\frac{1}{8\beta }{\left[\alpha -\beta \left(c_{m}+c_{s}\right)+B_{1}k_{0}\right]}^{2}}{\frac{1}{4\beta }{\left[\alpha -\beta \left(c_{m}+c_{s}\right)+B_{1}k_{0}\right]}^{2}-Ik_{0}^{2}} & \text{when} & I\leq \hat{I},\\ \frac{1}{4}\leq \varphi \leq \frac{1}{2} & \text{when} & \hat{I}<I<\bar{I},\\ \frac{\frac{1}{16\beta }{\left[\alpha -\beta \left(c_{m}+c_{s}\right)+B_{1}\left(k_{0}-N_{1}\right)\right]}^{2}-I{\left(k_{0}-N_{1}\right)}^{2}}{\frac{1}{4\beta }{\left[\alpha -\beta \left(c_{m}+c_{s}\right)+B_{1}\left(k_{0}-N_{1}\right)\right]}^{2}-I{\left(k_{0}-N_{1}\right)}^{2}}\leq \varphi \leq \frac{\frac{1}{8\beta }{\left[\alpha -\beta \left(c_{m}+c_{s}\right)+B_{1}\left(k_{0}-N_{1}\right)\right]}^{2}}{\frac{1}{4\beta }{\left[\alpha -\beta \left(c_{m}+c_{s}\right)+B_{1}\left(k_{0}-N_{1}\right)\right]}^{2}-I{\left(k_{0}-N_{1}\right)}^{2}} & \text{when} & I=\bar{I}. \end{array}$

*Also*, $\prod_{M/RIS}\left(p_{ris},\Delta k_{ris}\right)+\prod_{S/RIS}\left(w_{ris}\right)=\prod_{C}\left(p_{C},\Delta k_{C}\right)$.

The above result shows that the value of wholesale price of per unit raw material set by the supplier is lower than that of the unit cost of the supplier. Thus, this lower value of per unit wholesale price motivates the supplier to engage in revenue and investment-sharing (RIS) contract to generate some profits, which is sufficient to cover his cost. On the other hand, the manufacturer takes his optimal decisions, which are equivalent to the centralized decision. In addition, we see that, the total supply chain profit under RIS contract is equal to the centralized profit when the sharing proportion *φ* lies between the above ranges. Therefore, the contract perfectly coordinates the decentralized channel for certain values of *φ*. Furthermore, under this contract, both the supply chain players generate higher profits than in the decentralized case. Therefore, the contract is an efficient contract for the players as well as the supply chain.

Therefore, both the contracts are accepted by the supply chain entities, since the contract parameters provide a higher profit for each entity than the corresponding decentralized supply chain.

### 6.3 Numerical analysis

Since, comparison of the players’ profits pose some degree of analytical complexity, we resort to numerical analysis below. In what follows, we also conduct various numerical analyses to analyze the impact of problem parameters on the profit of the supply chain players and compare the results from two different coordination methods. Graphically we show the region $\hat{I}<I<\bar{I}$.

#### 6.3.1 Analyzing the impact of greening investment on profits

From our analytical results, we find that the greening investment has a significant effect on the strategic decisions of the players. Here we analyze the impact of greening investment parameter to understand which coordination method is preferable for the supply chain players and how their profits change.

**Fig 1:**
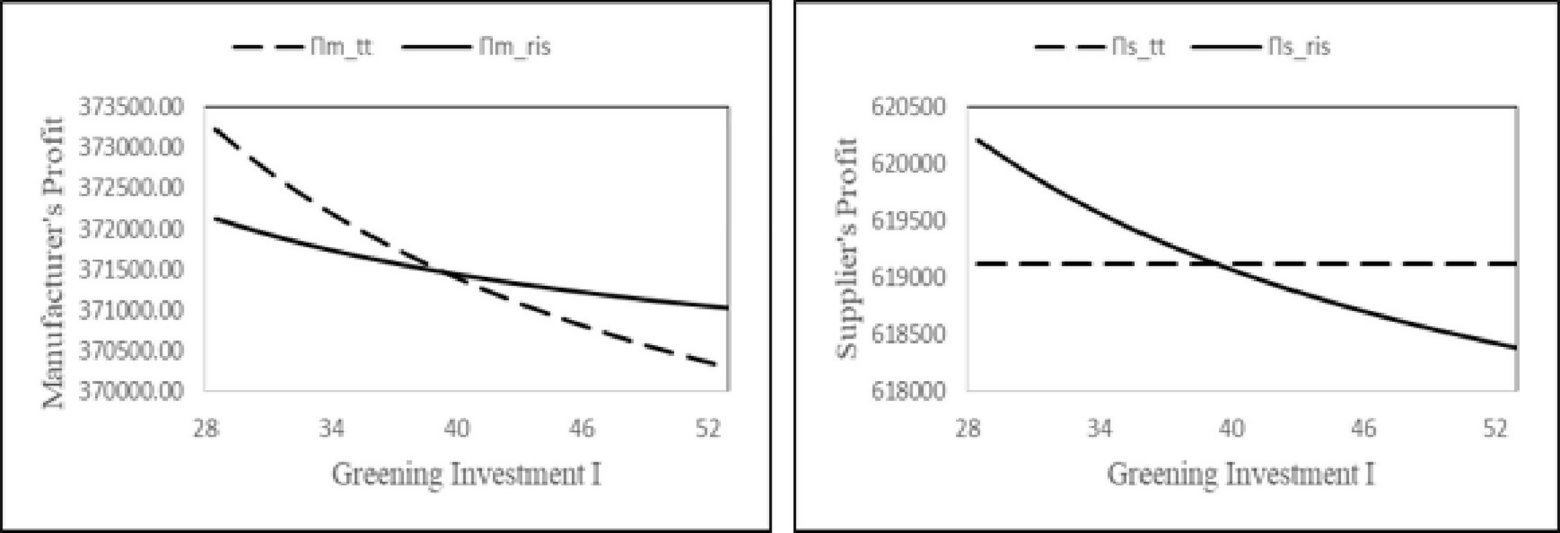
Comparison of manufacturer’s and supplier’s profit in two contracts w.r.t. the greening investment.

We consider the following values for the parameters: *α* = 1000, *β* = 0.25, *γ* = 0.8, *k*_0_ = 15, *N*_1_ = 11, *c*_*m*_ = 8, *c*_*s*_ = 20, *φ* = 0.375, *τ* = 1.5, and *F* = 620000 within the bounds of the problem parameters. The value of greening investment *I* is varied from 28 to 52 (refer to Fig 1). For expositional brevity, we present the figure for the above numerical parameters. The above figure illustrates the profits of the players in the region $\hat{I}<I<\bar{I}$ under the two different contracts. The results indicate that the profits of the players decrease with increase in greening investment cost. Since, the burden of carbon emission reduction falls on the manufacturer in both contracts (TT, RIS) and on the supplier in RIS contract only, the strategic decisions of the players are such that they tend to decrease overall supply chain profitability. Further, the result suggests that the manufacturer prefers the two-part tariff contract when the value of green investment parameter is low, whereas, for high values of this parameter, he prefers the revenue and investment-sharing contract. On the other hand, the choice of the supplier is opposite of the manufacturer. Since, the cost for greening investment shared by the supplier increases with the increase in value of this parameter, the RIS contract is desirable for the supplier for low values of greening investments. There are clear lessons for supply chain managers and policy makers. Policy makers should offer incentives to reduce cost burden of manufacturers besides establishing carbon emission norms.

#### 6.3.2 Analyzing the impact of price elasticity on profits

**Fig 2:**
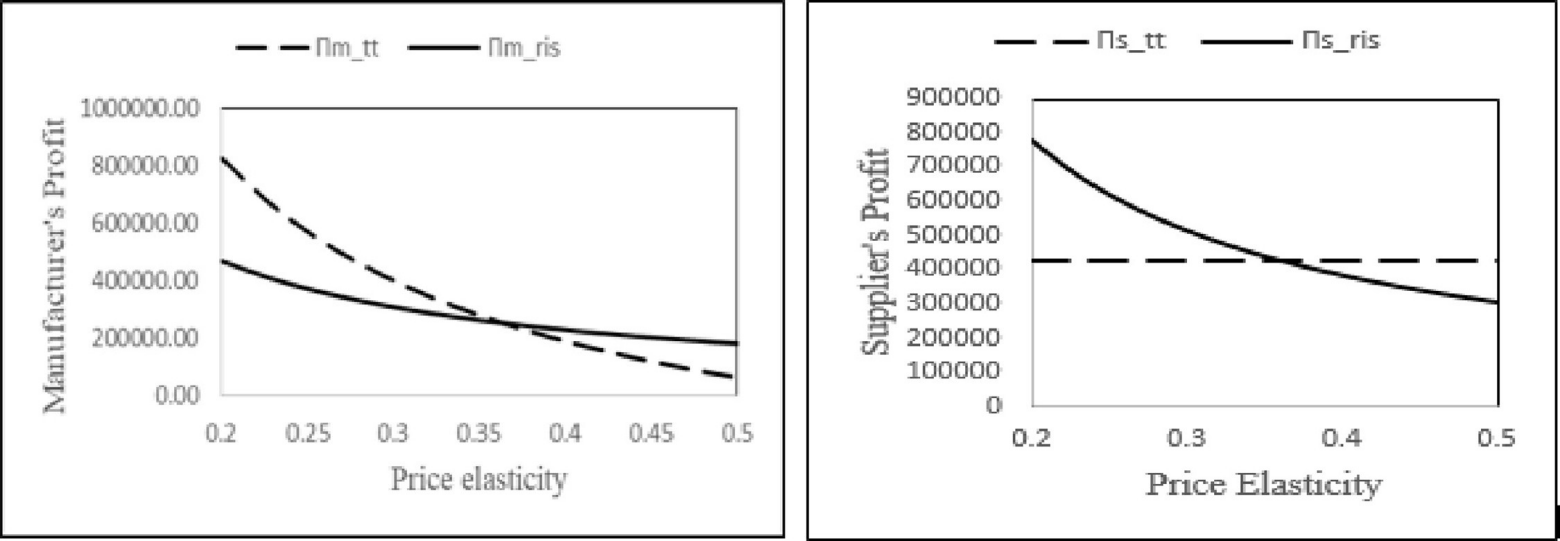
Comparison of manufacturer’s and supplier’s profit in two contracts w.r.t. the price elasticity(*β*).

To describe the effect of price elasticity on profits, we consider the following values for the parameters: *α* = 1000, *I* = 75, *γ* = 1, *k*_0_ = 15, *N*_1_ = 11, *c*_*m*_ = 8, *c*_*s*_ = 20 and *τ* = 0.6. The value of price elasticity *β* is varied from 0.2 to 0.5 (refer Fig 2).

The results show that the profits of the players and the supply chain decrease with increase in price sensitivity. Further, in Proposition 1(b) we have noted that the increase in price sensitivity results in the manufacturer reducing the amount of carbon reduction. As a result, the players’ profitability and the overall supply chain profits reduce. Clearly, price sensitive consumer markets are an impediment to carbon reduction initiatives. Efforts by policy makers to influence consumers to purchase green products, such as providing tax benefits, will significantly support the manufacturer to undertake some initiatives for reduction of carbon emission. In addition, from the perspective of coordination mechanisms, we observe that the two-part tariff and RIS contracts are preferable for the manufacturer and supplier respectively when consumers are less price sensitive. On the other hand, in a high price sensitive consumer market, the RIS contract is better for manufacturer whereas, the supplier prefers the two-part tariff contract.

#### 6.3.3 Analyzing the impact of carbon emission reduction elasticity on profits

**Fig 3:**
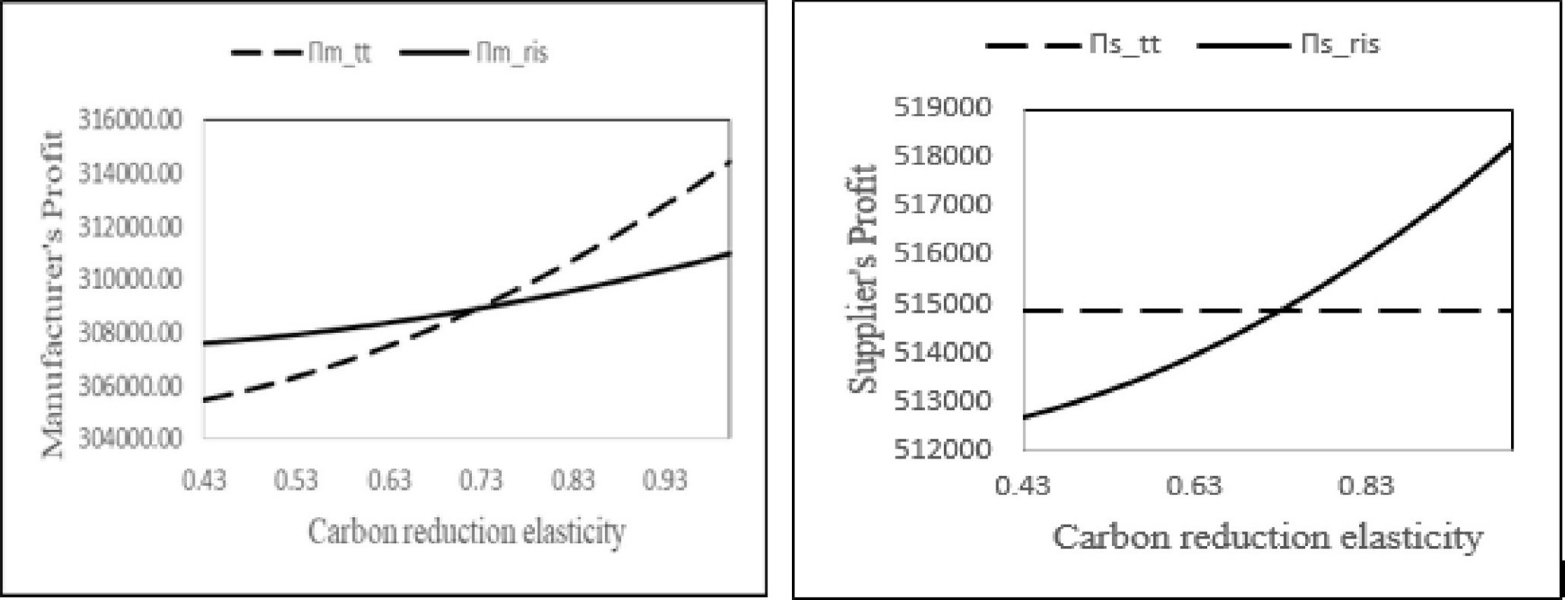
Comparison of manufacturer’s and supplier’s profit in two contracts w.r.t. the carbon reduction elasticity (*γ*).

To describe the effect of carbon emission reduction elasticity on profits, we consider the following parameter values:*α* = 1000, *I* = 47, *β* = 0.3, *k*_0_ = 15, *N*_1_ = 11, *c*_*m*_ = 8, *c*_*s*_ = 20 and *τ* = 0.6. The value of carbon reduction elasticity *γ* is varied between 0.43 to 1 (refer Fig 3).

We observe that the green sensitive consumer markets lead to higher profits for the players and the overall supply chain. Thus, policy makers and supply chain managers should undertake initiatives to incentivize consumers to purchase green products. Further, we see that the manufacturer prefers the RIS contract and supplier prefers the two-part tariff contract when environmental awareness of consumers is less. On the other hand, in the high green sensitive consumer market, the RIS is preferable for supplier whereas, the manufacturer prefers the two-part tariff contract.

In summary, we observe that a preference divergence emerges between the manufacturer and supplier depending on the market characteristics and greening costs in driving carbon reduction efforts. Depending on carbon emission reduction elasticity, price elasticity and coefficient of greening investment, the manufacturer and supplier prefer different contract types. A key lesson to draw for managers is to observe the market characteristics prior to accepting contracts with their supply chain partners. We discuss these in further detail in the next section.

#### 6.3.4 Insights for practice

From the above analysis, we derive certain key insights. *Tables 3–5 are true only for the region $\hat{I}<I<\bar{I}$.* We find that those markets which require higher investment costs for greening or have higher price sensitivity, the manufacturer prefers a revenue and investment sharing contract. For markets in which the investment cost is low or price sensitivity is low, the manufacturer prefers a two-part tariff contract. The intuition behind the result is as follows. A RIS contract provides the incentive of green cost sharing with the supplier which is crucial for the manufacturer under externalities such as high investment costs or high price sensitivity. Recall that under both these cases the manufacturer lowers his per unit carbon emission reduction (refer Propositions 1 and 2) that lowers market demand and his profits. Therefore, the RIS contract is his preferable contract. When the market characteristics are such that greening requires lower investment or consumers are less price sensitive, the manufacturer can provide higher carbon emission reduction effort and charge higher prices.

**Table 3:** Contract preferences of manufacturer and supplier at low and high values of I.

		Manufacturer Preference	Supplier Preference
**Greening Investment (I)**	**Low**	Two-part tariff contract	RIS contract
	**High**	RIS contract	Two-part tariff contract

**Table 4:** Contract preferences of manufacturer and supplier at low and high value of *β*.

		Manufacturer Preference	Supplier Preference
**Price sensitivity Parameter (*β*)**	**Low**	Two-part tariff contract	RIS contract
	**High**	RIS contract	Two-part tariff contract

**Table 5:** Contract preferences of manufacturer and supplier at low and high value of *γ*.

		Manufacturer Preference	Supplier Preference
**Sensitivity of Carbon Reduction (*γ*)**	**Low**	RIS contract	Two-part tariff contract
	**High**	Two-part tariff contract	RIS contract

In such a case, he prefers a contract which lowers his procurement cost from the supplier as enabled by the two-part tariff contract. The converse holds true for the supplier. Since under externalities such as higher investment costs, the supplier shares the cost of greening with the manufacturer, his profits are lower. Therefore, he prefers a two-part tariff contract in comparison to the RIS contract. The players’ preferences are summarized in Tables 3 and 4.

Next, when the carbon reduction sensitivity is higher in markets, we find that the manufacturer prefers the two-part tariff contract compared to the case when its lower (where the manufacturer prefers a RIS contract). The intuition is similar to the case above. When markets display higher positive externality such as carbon reduction sensitivity, the manufacturer increases his unit carbon reduction and charges higher prices (refer Proposition 1). He, therefore, prefers a contract which reduces his procurement cost with the supplier as provided by the two-part tariff contract. The converse holds true for the supplier. The result is summarized in Table 5.

Supply chain managers would therefore note that suitable incentives can be designed to drive efficient supply chain outcomes. Further, the results show that suppliers and manufacturers can consider different contracts based on market characteristics and greening costs in driving carbon reduction efforts.

From a policy perspective, we note that emission norms drive the carbon reduction efforts of manufacturers as desired (refer Proposition 3). However, stricter carbon emission norms increase selling price as the manufacturer transfers the higher procurement cost and cost of greening to the consumer. This reduces consumer welfare. Therefore, a social planner may consider subsidizing the emission cost of the manufacturer or influence consumer markets to support green manufacturers to influence demand (as we note in the case of vehicle manufacturers in the EU).

## 7. Discussion and conclusion

Carbon emissions norms are increasingly being used by policy makers globally to drive product greening initiatives. This, however, may lead to incentive conflict since the costs of adhering to regulations is borne by the manufacturers. In this the context, we consider a supply chain where the carbon cap applies to the manufacturer, who undertakes greening investments to reduce carbon emission of the product while procuring raw material from the supplier.

We analyze the strategic decisions of the supply chain players and study the impact of various parameters on these decisions. We examine various contracts to coordinate the decentralized supply chain under carbon emissions cap. Our analysis demonstrates that the manufacturer strategically decreases the level of carbon reduction in a price sensitive consumer market, however, product greening enables the manufacturer to charge a higher selling price of the product. The study also shows that the manufacturer decreases the amount of carbon reduction and selling price of the product with an increase in green investment cost to maintain his profits. We determine regions based on the investment coefficient of the greening initiative of the manufacturer and derive optimal carbon emission reduction decisions. We find that with increasing carbon reduction elasticity, the region in which the manufacturer provides more greening than mandated by government cap, increases. On the other hand, with increasing price sensitivity of customers, the region in which the manufacturer provides more greening than mandated by government cap decreases. The finding further suggests that stricter carbon emission standards force the manufacturer to increase the amount of carbon reduction and selling price.

However, support by partners in the supply chain, namely, the supplier, can help reduce the cost burden for the manufacturer. Therefore, we propose a two-part tariff contract, and a revenue and investment-sharing contract to coordinate the supply chain. We find that both the contracts perfectly coordinate the decentralized chain. Our numerical results illustrate the impact of key parameters such as greening investment cost, price sensitivity, and carbon reduction elasticity on individual players and compare the supply chain players’ profits under two different contracts. We find that based on the market characteristics and greening costs, the preference of contract for both the supplier and manufacturer varies.

In markets that require higher investment costs for greening or have higher price sensitivity, the manufacturer prefers a revenue and investment sharing contract whereas in markets in which the investment cost is low or price sensitivity is low, the manufacturer prefers a two-part tariff contract. Additionally, when the carbon reduction sensitivity is higher, the manufacturer prefers the two-part tariff contract whereas, when its lower, the manufacturer prefers a RIS contract. The above results have important implications for managers to design suitable incentives that can drive efficient supply chain outcomes. Our results also provide critical insights for social planners or regulators. We find that under stricter carbon emission norms the manufacturer increases the selling price to transfer the higher procurement cost and cost of greening to the consumer. This reduces consumer welfare. In these circumstances, a social planner may consider subsidizing the emission cost of the manufacturer or influence consumer markets to support green manufacturers.

### 7.1 Future extensions

Our study looks at a vertical structure consisting of a supplier and a manufacturer. This could be further extended to consider multiple manufacturers and suppliers and horizonal competition between them. Such an approach will provide an industry perspective when policy changes such as emissions cap apply. Secondly, future work could also consider a strategic dynamic decision making of firms over multiple periods while facing government-mandated carbon emissions cap. For example, a firm could be polluting in period 1, and the emissions cap implemented at the end of period 1, affects the firm in period 2. Lastly, we have considered the case of full information between the supply chain entities. This could be further extended to the case when there is information asymmetry between the partners to develop richer insights.

## Supporting information

S1 Appendix (DOCX)
