# Supplementary material for: Incentive conflict and supply contracts under carbon cap policy
Source: PLoS One. 2022 Nov 17;17(11):e0277777. doi: 10.1371/journal.pone.0277777 (PMC9671477; doi:10.1371/journal.pone.0277777)
Supplement: S1 Appendix — (DOCX) [file pone.0277777.s001.docx]

**S1 Appendix**

**Appendix A**

**Proof of lemma 1:**

| $\prod_{M}\left(p,\Delta k\right)$ | | $=$ | $\left(p-c_{m}-w-\tau\left(\Delta k\right)\right)D\left(p,\Delta k\right)-I\left(\Delta k\right)^{2}$ |  |
| --- | --- | --- | --- | --- |
| *s.t.,* | $\Delta k$ | $\geq$ | $k_{0}-N_{1}$ | (A.1) |

The corresponding Lagrangian function is given by

$$L\left( p,\Delta k,\lambda\right)=\left( p-c_{m}-w-\tau(\Delta k) \right)\left( \alpha-\beta p+\gamma\left( \Delta k \right) \right)-I\left( \Delta k \right)^{2}-\lambda\left( \left( \Delta k \right)-k_{0}+N_{1}-S^{2} \right)$$

Where $\lambda\left( \leq0 \right)$ is a Lagrange multiplier. The KKT necessary conditions are as follows:

| $\frac{\partial L}{\partial p}$ | $=-2\beta p+\left( \gamma+\beta\tau\right)\Delta k+\alpha+\beta\left( c_{m}+w \right)=0$ | (A.2) |
| --- | --- | --- |
| $\frac{\partial L}{\partial\Delta k}$ | $=\left( \gamma+\beta\tau\right)p-2\left( \tau\gamma+I \right)\Delta k-\tau\alpha-\gamma\left( c_{m}+w \right)-\lambda=0$ | (A.3) |
|  | $\lambda\left( \Delta k-k_{0}+N_{1} \right)=0$ and $\Delta k\geq k_{0}-N_{1}$, $\lambda\leq0, p,\Delta k\geq0$. | |

**Case 1:** **Case 1:** $\lambda=0$

$\lambda=0$ suggests that $\left( \Delta k-k_{0}+N_{1} \right)\neq0 i.e., \Delta k>k_{0}-N_{1}$.

If we substitute $\lambda=0$ in equations (A.2) & (A.3), we get,

$-2\beta p+\left( \gamma+\beta\tau\right)\Delta k+\alpha+\beta\left( c_{m}+w \right)=0$ and

$\left( \gamma+\beta\tau\right)p-2\left( \tau\gamma+I \right)\Delta k-\tau\alpha-\gamma\left( c_{m}+w \right)=0$.

Solving the above two equations, we get,

$\Delta k=\frac{B_{1}}{4\beta I-B_{1}^{2}}\left( \alpha-\beta\left( c_{m}+w \right) \right)$ and

$p=\frac{1}{4\beta I-B_{1}^{2}}\left[ 2\beta I \left( \frac{\alpha}{\beta}+c_{m}+w \right)\alpha+B_{1}\gamma\left( \frac{\alpha}{\beta}-c_{m}-w \right)-B_{1}^{2}\frac{\alpha}{\beta} \right]$

Since $\Delta k$ cannot exceed the initial carbon emission$\left( k_{0} \right)$, therefore, $\Delta k^{*}=Min \left\{ \Delta k,k_{0} \right\}$. Thus, by using the bound of $\Delta k$ i.e., $k_{0}-N_{1}<\Delta k\leq k_{0}$ , we get the bounds of investment parameter $I$ as $\hat{I}\leq I<\underline{I}$. Where $\hat{I}=\frac{B_{1}}{4\beta k_{0}}\left[ \alpha-\beta\left( c_{m}+w \right)+B_{1}k_{0} \right]$ and $\underline{I}=\frac{B_{1}}{4\beta\left( k_{0}-N_{1} \right)}\left[ \alpha-\beta\left( c_{m}+w \right)+B_{1}\left( k_{0}-N_{1} \right) \right]$. Therefore, $\Delta k^{*}=\Delta k$ only when $\hat{I}<I<\underline{I}$ and $\Delta k^{*}=k_{0}$. The remaining part is the same as given in the appendix.

Substituting the values of $\Delta k^{*}$in equation (A.2), the corresponding values of the selling price are given by

| $p_{11}^{*}=\frac{1}{4\beta I-B_{1}^{2}}\left[2\beta I\left(\frac{\alpha}{\beta}+c_{m}+w\right)+B_{1}\gamma\left(\frac{\alpha}{\beta}-c_{m}-w\right)-B_{1}^{2}\frac{\alpha}{\beta}\right],$ | (A.6) |
| --- | --- |

| $p_{12}^{*}=\frac{1}{2\beta}\left[\alpha+\beta\left(c_{m}+w\right)+\left(\gamma+\beta\tau\right)k_{0}\right].$ | (A.7) |
| --- | --- |

The corresponding demand functions are given by

| $D_{11}^{*}=\frac{2\beta^{2}I}{4\beta I-B_{1}^{2}}\left(\frac{\alpha}{\beta}-c_{m}-w\right)$ | and | $D_{12}^{*}=\frac{1}{2}\left[\alpha+B_{1}k_{0}-\beta\left(c_{m}+w\right)\right].$ | (A.8) |
| --- | --- | --- | --- |

Therefore, the supplier problem in this case is as follows:

| $\mathop{Max}\limits_{w} \prod_{S}\left(w\right)=\left(w-c_{s}\right)D^{*}\left(w\right)$ |  |
| --- | --- |

The first and second order conditions are given by

| $\frac{d}{dw_{11}}\prod_{S}=\frac{2\beta^{2}I}{4\beta I-B_{1}^{2}}\left[-2w_{11}+\frac{\alpha}{\beta}-c_{m}+c_{s}\right]=0$ | | and | | $\frac{d^{2}}{dw_{11}^{2}}\prod_{S}=-4\frac{\beta^{2}I}{4\beta I-B_{1}^{2}}\leq0,$ | |
| --- | --- | --- | --- | --- | --- |
| $\frac{d}{dw_{12}}\prod_{S}=-\beta w_{12}+\frac{1}{2}\left[\alpha+B_{1}k_{0}-\beta\left(c_{m}-c_{s}\right)\right]=0$ | and | | $\frac{d^{2}}{dw_{12}^{2}}\prod_{S}=-\beta\leq0.$ | |  |

Thus, the supplier's profit function is concave in both the regions. Solving first order conditions, we get,

| $w_{11}^{*}=\frac{1}{2}\left(\frac{\alpha}{\beta}-c_{m}+c_{s}\right)$ | and | $w_{12}^{*}=\frac{1}{2\beta}\left[\alpha+B_{1}k_{0}-\beta\left(c_{m}-c_{s}\right)\right].$ | (A.9) |
| --- | --- | --- | --- |

Using (A.9), the equations (A.5), (A.6) and (A.7) becomes,

| $p_{11}^{*}=\frac{1}{4\beta I-B_{1}^{2}}\left[\beta I\left(3\frac{\alpha}{\beta}+c_{m}+c_{s}\right)+\frac{B_{1}\gamma}{2}\left(\frac{\alpha}{\beta}-c_{m}-c_{s}\right)-B_{1}^{2}\frac{\alpha}{\beta}\right],$ | (A.10) |
| --- | --- |
| $p_{12}^{*}=\frac{1}{4\beta}\left[3\alpha+\beta\left(c_{m}+c_{s}\right)+\left(3\gamma+\beta\tau\right)k_{0}\right],$ | (A.11) |
| $\Delta k_{11}^{*}=\frac{B_{1}}{2\left(4\beta I-B_{1}^{2}\right)}\left[\alpha-\beta\left(c_{m}+c_{s}\right)\right].$ | (A.12) |

Using (A.9), the boundary value of investment parameter becomes

$\hat{I}=\frac{B_{1}}{8\beta k_{0}}\left[\alpha-\beta\left(c_{m}+c_{s}\right)+2B_{1}k_{0}\right]$ and $\bar{I}=\frac{B_{1}}{8\beta\left(k_{0}-N_{1}\right)}\left[\alpha-\beta\left(c_{m}+c_{s}\right)+2B_{1}\left(k_{0}-N_{1}\right)\right]$

**Case 2:**.$\lambda\neq0$

$\lambda\neq0$ suggests that $\left( \Delta k-k_{0}+N_{1} \right)=0 i.e., \Delta k^{*}=k_{0}-N_{1}$. Substituting $\Delta k^{*}=k_{0}-N_{1}$ in equations (A.1) & (A.2) and solved, we get ,

$p^{*}=\frac{1}{2\beta}\left[ \left( \gamma+\beta\tau\right)\left( k_{0}-N_{1} \right)+\alpha+\beta\left( c_{m}+w \right) \right]$ and $\lambda^{*}=\frac{1}{2\beta}\left[ -\left( 4\beta I-B_{1}^{2} \right)\left( k_{0}-N_{1} \right)+B_{1}\left( \alpha-\beta\left( c_{m}+w \right) \right) \right]$.

Using these solutions, the demand function can be written as

| $D_{2}^{*}=\frac{1}{2}\left[\alpha-\beta\left(c_{m}+w\right)+B_{1}\left(k_{0}-N_{1}\right)\right].$ | (A.14) |
| --- | --- |

Therefore, the supplier problem in this case is as follows:

| $\mathop{Max}\limits_{w} \prod_{S}\left(w\right)=\frac{1}{2}\left[\alpha-\beta\left(c_{m}+w\right)+B_{1}\left(k_{0}-N_{1}\right)\right]\left(w-c_{s}\right).$ | (A.15) |
| --- | --- |

The first and second order conditions are given by

| $\frac{d}{dw}\prod_{S}=\frac{1}{2}\left[-2\beta w+\alpha-\beta\left(c_{m}-c_{s}\right)+B_{1}\left(k_{0}-N_{1}\right)\right]$ | and | $\frac{d^{2}}{dw^{2}}\prod_{S}=-\beta\leq0.$ |
| --- | --- | --- |

Thus, the supplier's profit function is concave in$w$. Equating the first order condition to zero, we get,

| $w_{2}^{*}=\frac{1}{2}\left(\frac{\alpha}{\beta}-c_{m}+c_{s}+\frac{B_{1}}{\beta}\left(k_{0}-N_{1}\right)\right).$ | (A.16) |
| --- | --- |

Substituting the above value of $w$into the equations (A.13), we get,

| $p_{2}^{*}=\frac{1}{4\beta}\left[3\alpha+\beta\left(c_{m}+c_{s}\right)+\left(3\gamma+\beta\tau\right)\left(k_{0}-N_{1}\right)\right].$ | (A.17) |
| --- | --- |

This completes the proof of Lemma 1.

**Appendix B**

**Proof of lemma 2:** *The proof of Lemma 2 follows a similar approach.*

**Appendix C**

**Proof of positive selling price in Lemma 1:**

From Lemma 1, the optimal values of selling price under three different regions are given below:

$$p^{*}=\{3\alpha+\beta\left( c_{m}+c_{s} \right)+\left( 3\gamma+\beta\tau\right)k_{0} if I\leq\hat{I} \frac{1}{4\beta I-B_{1}^{2}}\left[ 4\beta I\left( 3\alpha+\beta\left( c_{m}+c_{s} \right) \right)+2B_{1}\gamma\left( \alpha-\beta\left( c_{m}+c_{s} \right) \right)-4B_{1}^{2}\alpha\right] 3\alpha+\beta\left( c_{m}+c_{s} \right)+\left( 3\gamma+\beta\tau\right)\left( k_{0}-N_{1} \right) if I=\underline{I} if \hat{I}\leq I\leq\underline{I}$$

It is observed that $3\alpha+\beta\left( c_{m}+c_{s} \right)+\left( 3\gamma+\beta\tau\right)k_{0}>0$ holds as per our problem assumptions. Thus the selling price is strictly positive in case 1.

In the second case, the optimal demand is $D_{11}^{*}=\frac{\beta I}{4\beta I-B_{1}^{2}}\left( \alpha-\beta\left( c_{m}+c_{s} \right) \right)>0$. Also, $4\beta I-B_{1}^{2}>0$ for the manufacturer's profit function to be concave. Therefore $\left( \alpha-\beta\left( c_{m}+c_{s} \right) \right)>0$ holds for positive demand. Note that the optimum value of marginal revenue of manufacturer is given by $p_{11}^{*}-c_{m}-w^{*}-\tau\left( \Delta k_{11}^{*} \right)=\frac{I}{4\beta I-B_{1}^{2}}\left( \alpha-\beta\left( c_{m}+c_{s} \right) \right)>0$. Therefore, the selling price is greater than zero.

In the third case, $\left( k_{0}-N_{1} \right)>0$ as per our problem assumption. Thus, the selling price in case 3 is greater than zero.

Proof of positive selling price in Lemma 2 follows a similar approach of lemma 1

**Appendix D**

**Proof of Proposition 1:**

(a) Differentiating both sides of (A.10), (A.11) and (A.17) w.r.t. $\gamma$ , we get,

$\frac{\partial p_{11}^{*}}{\partial\gamma}$ $=\frac{\left(4\beta I\gamma+B_{1}^{2}\beta\tau\right)}{2\left(4\beta I-B_{1}^{2}\right)^{2}}\left(\frac{\alpha}{\beta}-c_{m}-c_{s}\right)\geq0$,$\frac{\partial p_{12}^{*}}{\partial\gamma}=\frac{3}{4\beta}k_{0}\geq0$ and $\frac{\partial p_{2}^{*}}{\partial\gamma}=\frac{3}{4\beta}\left(k_{0}-N_{1}\right)\geq0.$

(b) Differentiating both sides of (A.12) w.r.t. $\beta$ , we get,

| $\frac{\partial\Delta k_{11}^{*}}{\partial\beta}$ | $=-\frac{1}{2\left(4\beta I-B_{1}^{2}\right)^{2}}\left(4I\gamma+B_{1}^{2}\tau\right)\left(\alpha-\beta\left(c_{m}+c_{s}\right)\right)-\frac{B_{1}}{2\left(4\beta I-B_{1}^{2}\right)}\left(c_{m}+c_{s}\right)\leq0$. |
| --- | --- |

**Appendix E**

**Proof of Proposition 2:** Differentiating both sides of (A.12) w.r.t.$I$ , we get,

| $\frac{\partial\Delta k_{11}^{*}}{\partial I}$ | $=-\frac{4\beta B_{1}\left(\alpha-\beta\left(c_{m}+c_{s}\right)\right)}{2\left(4\beta I-B_{1}^{2}\right)^{2}}$ | $=-\frac{2\beta B_{1}\left(\alpha-\beta\left(c_{m}+c_{s}\right)\right)}{\left(4\beta I-B_{1}^{2}\right)^{2}}$ | $\leq0$ |  |
| --- | --- | --- | --- | --- |

Differentiating both sides of (A.10) w.r.t.$I$ , we get,

| $\frac{\partial p_{11}^{*}}{\partial I}$ | $=-\frac{B_{1}}{\left(4\beta I-B_{1}^{2}\right)^{2}}\left(\gamma+\beta\tau\right)\left[\alpha-\beta\left(c_{m}+c_{s}\right)\right]\leq0$. |
| --- | --- |

**Appendix F**

**Proof of Proposition 3:** Differentiating both sides of (A.19), (A.17) and (A.18) w.r.t. $\beta$, we get,

$\frac{\partial\Delta k_{2}^{*}}{\partial N_{1}}=-1\leq0$, $\frac{\partial w_{2}^{*}}{\partial N_{1}}=-\frac{1}{2\beta}B_{1}\leq0$and $\frac{\partial p_{2}^{*}}{\partial N_{1}}=-\frac{1}{4\beta}\left(3\gamma+\beta\tau\right)\leq0.$

**Appendix G**

**Proof of Proposition 4:**

**Centralized Profit calculation:** In the region $\hat{I}<I<\bar{I}$, the optimal demand is given by

$D_{11}^{C}=\alpha-\beta p_{11}^{c}+\gamma\Delta k_{11}^{C}=\frac{2\beta I}{4\beta I-B_{1}^{2}}\left( \alpha-\beta\left( c_{m}+c_{s} \right) \right)$, Using (B.5) and (B.6)

| $p_{11}^{C}-c_{m}-c_{s}-\tau\left( \Delta k_{11}^{C} \right)$ | $=\frac{1}{\left( 4\beta I-B_{1}^{2} \right)\beta}\left[ 2\beta I\left( \alpha+\beta\left( c_{m}+c_{s} \right) \right)+B_{1}\gamma\left( \alpha-\beta\left( c_{m}+c_{s} \right) \right)-B_{1}^{2}\alpha\right]-\left( c_{m}+c_{s} \right)-\tau\frac{B_{1}}{\left( 4\beta I-B_{1}^{2} \right)}\left[ \alpha-\beta\left( c_{m}+c_{s} \right) \right]$ |
| --- | --- |
|  | $=\frac{2I}{4\beta I-B_{1}^{2}}\left[ \alpha-\beta\left( c_{m}+c_{s} \right) \right]$ |

Therefore, the total profit in the centralized channel in the region $\hat{I}<I<\bar{I}$ is given by

| $\Pi_{C}$ | $=\frac{4\beta I^{2}}{\left( 4\beta I-B_{1}^{2} \right)^{2}}\left[ \alpha-\beta\left( c_{m}+c_{s} \right) \right]^{2}-\frac{B_{1}^{2}I}{\left( 4\beta I-B_{1}^{2} \right)^{2}}\left[ \alpha-\beta\left( c_{m}+c_{s} \right) \right]^{2}$ | |
| --- | --- | --- |
|  | $=\frac{2I}{\left( 4\beta I-B_{1}^{2} \right)^{2}}\left[ \alpha-\beta\left( c_{m}+c_{s} \right) \right]^{2}$ | (p.1) |

Similarly, in the region $I\leq\hat{I}$

$D_{12}^{C}=\frac{1}{2}\left( \alpha-\beta\left( c_{m}+c_{s} \right)+B_{1}k_{0} \right)$, $p_{12}^{C}-c_{m}-c_{s}-\tau\left( \Delta k_{12}^{C} \right)=\frac{1}{2\beta}\left[ \alpha-\beta\left( c_{m}+c_{s} \right){+B_{1}k}_{0} \right]$

Therefore, the centralized profit in the region $I\leq\hat{I}$ is given by

|  | $=\frac{1}{2\beta}\left[\alpha-\beta\left(c_{m}+c_{s}\right)+B_{1}k_{0}\right]^{2}-Ik_{0}^{2}$ | (p.2) |
| --- | --- | --- |

Similarly at $I=\underline{I}$, we get,

$D_{2}^{C}=\frac{1}{2}\left( \alpha-\beta\left( c_{m}+c_{s} \right)+B_{1}\left( k_{0}-N_{1} \right) \right)$, $p_{2}^{C}-c_{m}-c_{s}-\tau\left( \Delta k_{2}^{C} \right)=\frac{1}{2\beta}\left[ \alpha-\beta\left( c_{m}+c_{s} \right)+B_{1}\left( k_{0}-N_{1} \right) \right]$

Therefore, the centralized channel’s profit at $I=\bar{I}$ is given by

|  | $=\frac{1}{2\beta}\left[\alpha-\beta\left(c_{m}+c_{s}\right)+B_{1}\left(k_{0}-N_{1}\right)\right]^{2}-I\left(k_{0}-N_{1}\right)^{2}$ | (p.3) |
| --- | --- | --- |

**Decentralized Profit Calculation:** In the region $\hat{I}<I<\bar{I}$, the optimal demand is given by

$D_{11}^{*}=\alpha-\beta p_{11}^{*}+\gamma\Delta k_{11}^{*}=\frac{\beta I}{4\beta I-B_{1}^{2}}\left( \alpha-\beta\left( c_{m}+c_{s} \right) \right)$ and

| $p_{11}^{*}-c_{m}-w^{*}-\tau\left( \Delta k_{11}^{*} \right)$ | $=\frac{1}{\left( 4\beta I-B_{1}^{2} \right)\beta}\left[ \beta I\left( 3\alpha+\beta\left( c_{m}+c_{s} \right) \right)+\frac{B_{1}\gamma}{2}\left( \alpha-\beta\left( c_{m}+c_{s} \right) \right)-B_{1}^{2}\alpha\right]-\frac{1}{2\beta}\left( {\alpha+\beta(c}_{m}+c_{s}) \right)-\tau\frac{B_{1}}{2\left( 4\beta I-B_{1}^{2} \right)}\left[ \alpha-\beta\left( c_{m}+c_{s} \right) \right]$ |
| --- | --- |
|  | $=\frac{I}{4\beta I-B_{1}^{2}}\left[ \alpha-\beta\left( c_{m}+c_{s} \right) \right]$ |

Therefore, the manufacturer’s profit in the region $\hat{I}<I<\bar{I}$ is given by

| $\Pi_{M}^{*}$ | $=\frac{\beta I^{2}}{\left( 4\beta I-B_{1}^{2} \right)^{2}}\left[ \alpha-\beta\left( c_{m}+c_{s} \right) \right]^{2}-\frac{B_{1}^{2}I}{{4\left( 4\beta I-B_{1}^{2} \right)}^{2}}\left[ \alpha-\beta\left( c_{m}+c_{s} \right) \right]^{2}$ | |
| --- | --- | --- |
|  | $=\frac{I}{4\left( 4\beta I-B_{1}^{2} \right)^{2}}\left[ \alpha-\beta\left( c_{m}+c_{s} \right) \right]^{2}$ |  |

and

The supplier’s profit is given by

|  | $=\left(w^{*}-c_{s}\right)\frac{\beta I}{4\beta I-B_{1}^{2}}\left[\alpha-\beta\left(c_{m}+c_{s}\right)\right]=\frac{I}{2\left(4\beta I-B_{1}^{2}\right)}\left[\alpha-\beta\left(c_{m}+c_{s}\right)\right]^{2}$ |  |
| --- | --- | --- |

Therefore, the total supply chain’s profit is given by

$\Pi_{DC}^{*}=\frac{I}{4\left( 4\beta I-B_{1}^{2} \right)^{2}}\left[ \alpha-\beta\left( c_{m}+c_{s} \right) \right]^{2}$ (p.4)

From (p.1) and (p.2), we get,

$\frac{4}{3}\prod_{DC}^{*}=\prod_{C}^{*}$

Similarly, in the region $I\leq\hat{I}$

$D_{12}^{*}=\frac{1}{4}\left( \alpha-\beta\left( c_{m}+c_{s} \right)+B_{1}k_{0} \right)$, $p_{12}^{*}-c_{m}-c_{s}-\tau\left( \Delta k_{12}^{*} \right)=\frac{1}{4\beta}\left[ \alpha-\beta\left( c_{m}+c_{s} \right){+B_{1}k}_{0} \right]$

Therefore, the manufacturer’s and supplier’s profit in the region $I\leq\hat{I}$ is given by

$\Pi_{M}^{*}=\frac{I}{16\beta}\left[ \alpha-\beta\left( c_{m}+c_{s} \right)+B_{1}k_{0}) \right]^{2}-Ik_{0}^{2}$ and $\Pi_{S}^{*}=\frac{I}{8\beta}\left[ \alpha-\beta\left( c_{m}+c_{s} \right)+B_{1}k_{0}^{2}) \right]^{2}$

Therefore, the total supply chain’s profit in the decentralized channel is given by

$\Pi_{DC}^{*}=\frac{3}{16\beta}\left[ \alpha-\beta\left( c_{m}+c_{s} \right)+B_{1}k_{0} \right]^{2}-Ik_{0}^{2}$ (p.5)

From (p.2) and (p.5), we get,

$\frac{4}{3}\Pi_{DC}^{*}=\Pi_{C}^{*}-\frac{1}{3}Ik_{0}^{2}\leq\Pi_{C}^{*}$.

Similarly at $I=\underline{I}$, we get,

$D_{2}^{*}=\frac{1}{4}\left( \alpha-\beta\left( c_{m}+c_{s} \right)+B_{1}\left( k_{0}-N_{1} \right) \right)$, $p_{2}^{*}-c_{m}-c_{s}-\tau\left( \Delta k_{2}^{*} \right)=\frac{1}{4\beta}\left[ \alpha-\beta\left( c_{m}+c_{s} \right)+B_{1}\left( k_{0}-N_{1} \right) \right]$

Therefore, the manufacturer’s & supplier’s profit in the region at $I=\underline{I}$ is given by

$\Pi_{M}^{*}=\frac{I}{16\beta}\left[ \alpha-\beta\left( c_{m}+c_{s} \right)+B_{1}\left( k_{0}-N_{1} \right)) \right]^{2}-I\left( k_{0}-N_{1} \right)^{2}$ and $\Pi_{S}^{*}=\frac{I}{8\beta}\left[ \alpha-\beta\left( c_{m}+c_{s} \right)+B_{1}\left( k_{0}-N_{1} \right)^{2}) \right]^{2}$

Therefore, the total supply chain’s profit in decentralized channel is given by

$\Pi_{DC}^{*}=\frac{3}{16\beta}\left[ \alpha-\beta\left( c_{m}+c_{s} \right)+B_{1}\left( k_{0}-N_{1} \right) \right]^{2}-I\left( k_{0}-N_{1} \right)^{2}$ (p.6) (p.5)

From (p.3) and (p.5), we get,

$\frac{4}{3}\Pi_{DC}^{*}=\Pi_{C}^{*}-\frac{1}{3}I\left( k_{0}-N_{1} \right)^{2}\leq\Pi_{C}^{*}$.

**Appendix H**

**Proof of Proposition 5 :** The profits of the manufacturer and supplier are given by

| $\prod_{M/tt}\left(p,\Delta k\right)$ | $=$ | $\left(p-c_{m}-w_{tt}-\tau\Delta k\right)(\alpha\text{- }\beta p\text{+}\gamma\Delta\text{k})-I\left(\Delta k\right)^{2}-F_{tt}$ | |
| --- | --- | --- | --- |
| s.t., $\Delta k\geq k_{0}-N_{1}$ | | | |
| $\prod_{S/tt}\left(w\right)$ | $=$ | $\left(w_{tt}-c_{s}\right)\left(\alpha-\beta p+\gamma\Delta k\right)+F_{tt}$ |  |

The first order conditions are written as

| $\frac{\partial\prod_{M/tt}}{\partial p}=-2\beta p+\left(\gamma+\beta\tau\right)\Delta k+\alpha+\beta\left(c_{m}+w_{tt}\right)=0$ | (p.1) |
| --- | --- |
| $\frac{\partial\prod_{M/tt}}{\partial\Delta k}=\left(\gamma+\beta\tau\right)p-2\left(I+\gamma\tau\right)\Delta k-\tau\alpha-\gamma\left(c_{m}+w_{tt}\right)=0$ | (p.1) |

In the region$\hat{I}<I<\bar{I}$, substituting the centralized optimal values of decision variables in the equation (p.1) and simplifying the equations, we get,$w_{tt}=c_{s}$.

At $w_{tt}=c_{s}$, the profits of the manufacturer and supplier can be written as

| $\prod_{M/tt}\left(p,\Delta k\right)=\prod_{C}\left(p_{C},\Delta k_{C}\right)-F_{tt}$ and $\prod_{S/tt}=\left(w_{tt}-c_{s}\right)\left(\alpha-\beta p+\gamma\Delta k\right)+F_{tt}=F_{tt}$ |
| --- |

Both parties would agree to this contract when $\prod_{M/tt}\left(p_{tt},\Delta k_{tt}\right)\geq\prod_{M}^{*}$and$\prod_{\frac{S}{tt}}\left(w_{tt}\right)\geq\prod_{S}^{*}$. Thus, $\prod_{M/tt}\left(p_{tt},\Delta k_{tt}\right)\geq\prod_{M}^{*}$gives $\prod_{C}\left(p_{C},\Delta k_{C}\right)-F_{tt}\geq\prod_{M}^{*}$.

| Which is equivalent to | |
| --- | --- |
| $F_{tt}\leq$ $\frac{3}{4}\frac{I\left[\alpha-\beta\left(c_{m}+c_{s}\right)\right]^{2}}{\left(4\beta I-B^{2}\right)}$ | and $\prod_{S/tt}\left(w_{tt}\right)\geq\prod_{S}^{*}\Rightarrow F_{tt}\geq\frac{I\left[\alpha-\beta\left(c_{m}+c_{s}\right)\right]^{2}}{2\left(4\beta I-B^{2}\right)}$. |

Therefore, we can write, $\frac{I\left[\alpha-\beta\left(c_{m}+c_{s}\right)\right]^{2}}{2\left(4\beta I-B^{2}\right)}\leq F_{tt}\leq\frac{3}{4}\frac{I\left[\alpha-\beta\left(c_{m}+c_{s}\right)\right]^{2}}{\left(4\beta I-B^{2}\right)}$.

Similarly, in the region$I\leq\hat{I}\text{ and }I=\bar{I}$, the range of $F_{tt}$ are

| $\frac{1}{8\beta}\left[\alpha-\beta\left(c_{m}+c_{s}\right)+B_{1}k_{0}\right]^{2}\leq F_{tt}\leq$ $\frac{3}{16\beta}\left[\alpha-\beta\left(c_{m}+c_{s}\right)+B_{1}k_{0}\right]^{2}$and |  |
| --- | --- |

$\frac{1}{8\beta}\left[\alpha-\beta\left(c_{m}+c_{s}\right)+B_{1}\left(k_{0}-N_{1}\right)\right]^{2}\leq F_{tt}\leq$ $\frac{3}{16\beta}\left[\alpha-\beta\left(c_{m}+c_{s}\right)+B_{1}\left(k_{0}-N_{1}\right)\right]^{2}$ respectively.

**Appendix I**

**Proof of the proposition 6:** Proof (i): Under the revenue and investment-sharing contract, the problem of the manufacturer becomes

| $\prod_{M/RIS}\left(p,\Delta k\right)=\left(\phi p-c_{m}-w_{ris}-\phi\tau\Delta k\right)(\alpha\text{- }\beta p\text{+}\gamma\Delta\text{k})-\phi I\left(\Delta k\right)^{2}$ |  | |
| --- | --- | --- |
| *s.t.,* $\Delta k\geq k_{0}-N$ | |  |

The first order conditions are

| $\frac{\partial}{\partial p}\prod_{M/RIS}=-2\beta\phi p+\left(\gamma+\beta\tau\right)\phi\Delta k+\phi\alpha+\beta\left(c_{m}+w_{ris}\right)=0$ | (p.3) |
| --- | --- |
| $\frac{\partial}{\partial\Delta k}\prod_{M/RIS}=\left(\gamma+\beta\tau\right)\phi p-2\left(I+\gamma\tau\right)\phi\Delta k-\phi\tau\alpha-\gamma\left(c_{m}+w_{ris}\right)=0$ | (p.4) |

The second order derivatives are as follows

$$\frac{\partial^{2}}{\partial p^{2}}\prod_{M/RIS}=-2\beta\phi,\frac{\partial^{2}}{\partial\Delta k^{2}}\prod_{M/RIS}=-2\left(I+\gamma\tau\right)\phi,\frac{\partial^{2}}{\partial p\partial\Delta k}\prod_{M/RIS}=\left(\gamma+\beta\tau\right)=\frac{\partial^{2}}{\partial\Delta k\partial p}\prod_{M/RIS}$$

Therefore, the derivative of the hessian matrix is$\phi^{2}\left(4\beta I-B_{1}^{2}\right)\geq0$. Hence, the manufacturer profit function is jointly concave in$p\text{ and }\Delta k$.

Solving equations (p.14) and (p.15), we get,

| $p_{ris}=\frac{1}{\left(4\beta I-B_{1}^{2}\right)\phi}\left[2\beta I\left(\phi\frac{\alpha}{\beta}+c_{m}+w_{ris}\right)+B_{1}\gamma\left(\phi\frac{\alpha}{\beta}-c_{m}-w_{ris}\right)-B_{1}^{2}\phi\frac{\alpha}{\beta}\right],$ |  |
| --- | --- |
| $\Delta k_{ris}=\frac{B_{1}\beta}{\left(4\beta I-B_{1}^{2}\right)\phi}\left[\phi\frac{\alpha}{\beta}-c_{m}-w_{ris}\right]$ |  |

In the region$\hat{I}<I<\bar{I}$, this contract will coordinate if$p_{ris}=p_{C}\text{ and }\Delta\text{k}_{\text{ris}}=\Delta k_{C}$. Therefore,

| $\Delta\text{k}_{\text{ris}}=\Delta k_{C}$ | $\Rightarrow\frac{B_{1}\beta}{\left(4\beta I-B_{1}^{2}\right)\phi}\left[\phi\frac{\alpha}{\beta}-c_{m}-w_{ris}\right]=\frac{B_{1}\beta}{\left(4\beta I-B_{1}^{2}\right)}\left[\frac{\alpha}{\beta}-c_{m}-c_{s}\right]$ $\Rightarrow w_{ris}=\phi c_{s}-\left(1-\phi\right)c_{m}$ |
| --- | --- |

**Proof (ii):** Under this contract, the optimal profit of the manufacturer and supplier are given by

| $\prod_{M/RIS}=\frac{4\beta^{3}I^{2}}{\left(4\beta I-B_{1}^{2}\right)^{2}}\phi\left(\frac{\alpha}{\beta}-c_{m}-c_{s}\right)^{2}-\frac{\phi IB_{1}^{2}\beta^{2}}{\left(4\beta I-B_{1}^{2}\right)^{2}}\left(\frac{\alpha}{\beta}-c_{m}-c_{s}\right)^{2}=\frac{\phi\beta^{2}I}{\left(4\beta I-B_{1}^{2}\right)}\left(\frac{\alpha}{\beta}-c_{m}-c_{s}\right)^{2}=\phi\prod_{C}$ and |
| --- |
| $\prod_{S/RIS}=\left[\left(1-\phi\right)\left(p_{ris}-\tau\Delta k_{ris}\right)+w_{ris}-c_{s}\right]D_{ris}-\left(1-\phi\right)I\left(\Delta k_{ris}\right)^{2}=\frac{\left(1-\phi\right)\beta^{2}I}{\left(4\beta I-B_{1}^{2}\right)}\left(\frac{\alpha}{\beta}-c_{m}-c_{s}\right)^{2}=\left(1-\phi\right)\prod_{C}$ |

Both parties would agree to this contract when $\prod_{M/RIS}\left(p_{ris},\Delta k_{ris}\right)\geq\prod_{M}^{*}$and$\prod_{S/RIS}\left(w_{ris}\right)\geq\prod_{S}^{*}$.

Thus,

| $\prod_{M/RIS}\left(p_{ris},\Delta k_{ris}\right)\geq\prod_{M}^{*}\Rightarrow\phi\prod_{C}\geq\prod_{M}^{*}\Rightarrow\phi\geq\frac{\prod_{M}^{*}}{\prod_{C}}=\frac{1}{4}$ and | (p.5) |
| --- | --- |

| $\prod_{S/RIS}\left(w_{ris}\right)\geq\prod_{S}^{*}\Rightarrow\left(1-\phi\right)\prod_{C}\geq\prod_{S}^{*}\Rightarrow\left(1-\phi\right)\geq\frac{\prod_{S}^{*}}{\prod_{C}}=\frac{1}{2}\Rightarrow\phi\leq\frac{1}{2}$ | (p.6) |
| --- | --- |

Therefore, from (p.5) and (p.6), we can conclude that both parties would agree to this contract if $\phi$lies between$\left[\frac{1}{4},\frac{1}{2}\right]$.

Similarly, we get the range of $\phi$for other regions.

Table S1: Equilibrium Values under Decentralized Channel

| Equilibrium Values | Decentralized Channel | | |
| --- | --- | --- | --- |
|  | Case 1 (c1) | | Case 2 (c2) |
|  | Optimal Emission Case | Zero Emission Case |  |
| $\Delta k^{*}$ | $\frac{B_{1}}{2\left(4\beta I-B_{1}^{2}\right)}\left[\alpha-\beta\left(c_{m}+c_{s}\right)\right]$ | $k_{0}$ | $k_{0}-N_{1}$ |
| $p^{*}$ | $\frac{1}{4\beta I-B_{1}^{2}}\left[\beta I\left(3\frac{\alpha}{\beta}+c_{m}+c_{s}\right)\right.$ $\left.+\frac{B_{1}\gamma}{2}\left(\frac{\alpha}{\beta}-c_{m}-c_{s}\right)-B_{1}^{2}\frac{\alpha}{\beta}\right]$ | $\frac{1}{4\beta}\left[\begin{matrix} 3\alpha+\beta\left(c_{m}+c_{s}\right) \\ +\left(3\gamma+\beta\tau\right)k_{0} \end{matrix}\right]$ | $\frac{1}{4\beta}\left[\begin{matrix} 3\alpha+\beta\left(c_{m}+c_{s}\right) \\ +\left(3\gamma+\beta\tau\right)\left(k_{0}-N_{1}\right) \end{matrix}\right]$ |
| $w^{*}$ | $\frac{1}{2}\left[\frac{\alpha}{\beta}-c_{m}+c_{s}\right]$ | $\frac{1}{2\beta}\left[\begin{matrix} \alpha-\beta\left(c_{m}-c_{s}\right) \\ +B_{1}k_{0} \end{matrix}\right]$ | $\frac{1}{2\beta}\left[\begin{matrix} \alpha-\beta\left(c_{m}-c_{s}\right) \\ +B_{1}\left(k_{0}-N_{1}\right) \end{matrix}\right]$ |
|  | $\frac{I}{4\left(4\beta I-B_{1}^{2}\right)}\left[\alpha-\beta\left(c_{m}+c_{s}\right)\right]^{2}$ | $\begin{matrix} \frac{1}{16\beta}\left[\begin{matrix} \alpha-\beta\left(c_{m}+c_{s}\right) \\ +B_{1}k_{0} \end{matrix}\right]^{2} \\ -Ik_{0}^{2} \end{matrix}$ | $\begin{matrix} \frac{1}{16\beta}\left[\begin{matrix} \alpha-\beta\left(c_{m}+c_{s}\right) \\ +B_{1}\left(k_{0}-N_{1}\right) \end{matrix}\right]^{2} \\ -I\left(k_{0}-N_{1}\right)^{2} \end{matrix}$ |
|  | $\frac{I}{2\left(4\beta I-B_{1}^{2}\right)}\left[\alpha-\beta\left(c_{m}+c_{s}\right)\right]^{2}$ | $\begin{matrix} \frac{1}{8\beta}\left[\begin{matrix} \alpha-\beta\left(c_{m}+c_{s}\right) \\ +B_{1}k_{0} \end{matrix}\right]^{2} \\ \end{matrix}$ | $\frac{1}{8\beta}\left[\begin{matrix} \alpha-\beta\left(c_{m}+c_{s}\right) \\ +B_{1}\left(k_{0}-N_{1}\right) \end{matrix}\right]^{2}$ |
|  | $\frac{3I}{4\left(4\beta I-B_{1}^{2}\right)}\left[\alpha-\beta\left(c_{m}+c_{s}\right)\right]^{2}$ | $\begin{matrix} \frac{3}{16\beta}\left[\begin{matrix} \alpha-\beta\left(c_{m}+c_{s}\right) \\ +B_{1}k_{0} \end{matrix}\right]^{2} \\ -Ik_{0}^{2} \end{matrix}$ | $\begin{matrix} \frac{3}{16\beta}\left[\begin{matrix} \alpha-\beta\left(c_{m}+c_{s}\right) \\ +B_{1}\left(k_{0}-N_{1}\right) \end{matrix}\right]^{2} \\ -I\left(k_{0}-N_{1}\right)^{2} \end{matrix}$ |

Table S2: Equilibrium Values under Centralized Channel

| Equilibrium Values | Centralized Channel | | |
| --- | --- | --- | --- |
|  | Case 1 (c1) | | Case 2 (c2) |
|  | Optimal Emission Case | Zero Emission Case |  |
| $\Delta k_{C}$ | $\frac{B_{1}}{4\beta I-B_{1}^{2}}\left[\alpha-\beta\left(c_{m}+c_{s}\right)\right]$ | $k_{0}$ | $k_{0}-N_{1}$ |
| $p_{C}$ | $\frac{1}{4\beta I-B_{1}^{2}}\left[2\beta I\left(\frac{\alpha}{\beta}+c_{m}+c_{s}\right)\right.$ $\left.+B_{1}\gamma\left(\frac{\alpha}{\beta}-c_{m}-c_{s}\right)-B_{1}^{2}\frac{\alpha}{\beta}\right]$ | $\begin{matrix} \frac{1}{2\beta}\left[\alpha+\beta\left(c_{m}+c_{s}\right)\right. \\ \left.+\left(\gamma+\beta\tau\right)k_{0}\right] \end{matrix}$ | $\begin{matrix} \frac{1}{2\beta}\left[\alpha+\beta\left(c_{m}+c_{s}\right)\right. \\ \left.+\left(\gamma+\beta\tau\right)\left(k_{0}-N_{1}\right)\right] \end{matrix}$ |
|  | $\frac{I}{\left(4\beta I-B_{1}^{2}\right)}\left[\alpha-\beta\left(c_{m}+c_{s}\right)\right]^{2}$ | $\begin{matrix} \frac{1}{4\beta}\left[\begin{matrix} \alpha-\beta\left(c_{m}+c_{s}\right) \\ +B_{1}k_{0} \end{matrix}\right]^{2} \\ -Ik_{0}^{2} \end{matrix}$ | $\begin{matrix} \frac{1}{4\beta}\left[\begin{matrix} \alpha-\beta\left(c_{m}+c_{s}\right) \\ +B_{1}\left(k_{0}-N_{1}\right) \end{matrix}\right]^{2} \\ -I\left(k_{0}-N_{1}\right)^{2} \end{matrix}$ |
